# Geographic destiny trumps taxonomy in the Roundtail Chub, *Gila robusta* species complex (Teleostei, Leuciscidae)

**DOI:** 10.1038/s41598-023-41719-9

**Published:** 2023-09-22

**Authors:** Christopher R. Suchocki, Cassie Ka‘apu-Lyons, Joshua M. Copus, Cameron A. J. Walsh, Anne M. Lee, Julie Meka Carter, Eric A. Johnson, Paul D. Etter, Zac H. Forsman, Brian W. Bowen, Robert J. Toonen

**Affiliations:** 1 Hawaiʻi Institute of Marine Biology, University of Hawaiʻi at Mānoa, 46-007 Lilipuna Road, Kāneʻohe, HI 96744 USA; 2 Arizona Game and Fish Department, 5000 W. Carefree Highway, Phoenix, AZ 85086 USA; 3 Institute of Molecular Biology, University of Oregon, 1585 E 13th Ave., Eugene, OR 97403 USA; 4 Reefscape Restoration Initiative, King Abdullah University of Science and Technology (KAUST), Thuwal, 23955-6900 Kingdom of Saudi Arabia

**Keywords:** Ecology, Environmental sciences

## Abstract

The *Gila robusta* species complex in the lower reaches of the Colorado River includes three nominal and contested species (*G. robusta, G. intermedia,* and *G. nigra*) originally defined by morphological and meristic characters. In subsequent investigations, none of these characters proved diagnostic, and species assignments were based on capture location. Two recent studies applied conservation genomics to assess species boundaries and reached contrasting conclusions: an ezRAD phylogenetic study resolved 5 lineages with poor alignment to species categories and proposed a single species with multiple population partitions. In contrast, a dd-RAD coalescent study concluded that the three nominal species are well-supported evolutionarily lineages. Here we developed a draft genome (~ 1.229 Gbp) to apply genome-wide coverage (10,246 SNPs) with nearly range-wide sampling of specimens (*G. robusta* N = 266, *G. intermedia* N = 241, and *G. nigra* N = 117) to resolve this debate. All three nominal species were polyphyletic, whereas 5 of 8 watersheds were monophyletic. AMOVA partitioned 23.1% of genetic variance among nominal species, 30.9% among watersheds, and the Little Colorado River was highly distinct (*F*_*ST*_ ranged from 0.79 to 0.88 across analyses). Likewise, DAPC identified watersheds as more distinct than species, with the Little Colorado River having 297 fixed nucleotide differences compared to zero fixed differences among the three nominal species. In every analysis, geography explains more of the observed variance than putative taxonomy, and there are no diagnostic molecular or morphological characters to justify species designation. Our analysis reconciles previous work by showing that species identities based on type location are supported by significant divergence, but natural geographic partitions show consistently greater divergence. Thus, our data confirm *Gila robusta* as a single polytypic species with roughly a dozen highly isolated geographic populations, providing a strong scientific basis for watershed-based future conservation.

## Introduction

Freshwater ecosystems cover less than 1% of the planet’s surface yet harbor approximately half of the world’s fish diversity. Factors implicated in the high rate of speciation among freshwater fishes include productivity and isolation^1,2^. Glacial cycles, droughts, floods, stream captures, landslides, volcanic activity, tectonic uplifting, and even beaver dams can change stream geomorphology and lead to the isolation of freshwater bodies^3,4^. Glacial cycles through the Plio‐Pleistocene have been identified as a “species pump” for freshwater fishes in both North America^5^ and Australia^6^. With such a history of rapid radiations, freshwater fishes have become the focus of considerable research to understand speciation through the lens of genetic differentiation, with model systems such as Threespine Sticklebacks^7–10^, salmonids^11–14^, and African rift lake cichlids^15–18^.

In the lower reaches of the Colorado River of southwestern North America, substantial genetic differentiation has developed within the *Gila robusta* complex. Putative species from this group have undergone numerous taxonomic rearrangements (see Copus et al.^19^ for detailed taxonomic history). The three previously recognized species (*Gila robusta, G. intermedia,* and *G. nigra*) were originally defined by a few characters but none proved diagnostic, and species could only be identified by mean differences in meristic counts among localities^20–23^. However, traditional meristic counts and measurement differences do not hold up in the field, requiring statistical approaches such as discriminant function analyses or principal components of morphological variation^19–22,24^, and historical hybridization and anthropogenic movements of fishes have further muddled the phylogenetic framework for species assessments^25,26^. Species assignments are based on a combination of statistically defined differences anchored by drainage location because of overlap among morphological characters among the nominal taxa^20–22,24^. Given the lack of phylogenetic or morphologically diagnostic characters, the *Gila robusta* species complex was recently re-defined as a single polytypic unit leading to withdrawal of proposed “threatened” status under the U.S. Endangered Species Act (ESA) for *G. robusta* although no final action has been taken by USFWS for *G. intermedia* at this time^23,24,27^. The ESA status of this group is of considerable conservation interest because the Lower Colorado River Basin supplies nearly half of the municipal and agricultural water for the state of Arizona, creating tension between conservation goals and water usage^25,28^.

Despite the degree of attention afforded to this group, debate continues because previous studies came to conflicting conclusions about the taxonomy and conservation status of the *G. robusta* complex. For example, Copus et al.^19,24^ compiled a systematic and taxonomic review of the seven generic and fifteen specific names applied to these fishes, and applied ezRAD^29^ reduced-representation genomic data to support a single polytypic species with no diagnostic molecular characters to support the nominal taxonomy. Their data revealed 5 well resolved lineages, each containing more than one of the nominal species. Despite modest sampling, Copus et al.^19^ showed that the morphological variability within each of the nominal species precluded assigning fresh specimens to any type series. Indeed, different morphological characters (e.g., pectoral fin rays, upper procurrent caudal rays, and lateral line scales) can assign the same individual to multiple species. In contrast, Chafin et al.^25^ used ddRAD^30^ reduced-representation genomic data with larger sample sizes and SNP-based coalescence and polymorphism-aware phylogenetic models to argue the three nominal species are well-supported evolutionarily lineages, although with widespread phylogenetic discordance. Chafin et al.^25^ use a coalescent model testing framework to conclude that the lineages diverged during rapid Plio-Pleistocene drainage evolution, with subsequent divergence within the “anomaly zone”^31^ of tree space producing ambiguities that have confounded prior studies. Despite extensive geographic and genomic sampling, researchers reached conflicting conclusions about taxa in the *Gila robusta* complex. The water demand and commercial interests for the Lower Colorado River Basin, coupled with projected decreased water availability under future climate models, intensifies the scientific debate about how and why results differ among studies.

Here we undertake an extensive sampling of the geographic distribution of streams in which all members of the *G. robusta* complex are found. We use this extensive geographic and taxonomic sampling to perform hierarchical analyses comparing the relative effects of isolation among watersheds and nominal taxonomic designations to evaluate which hypothesis best explains the patterns of genetic structure observed in this region. By comparing genetic structure among watersheds and among species designations, we attempt to resolve how previous population genomic studies have come to differing conclusions and provide guidance on resource management for this complex of freshwater fishes.

## Results

### *Gila robusta* genome and nextRAD sequencing

**Figure 1:**
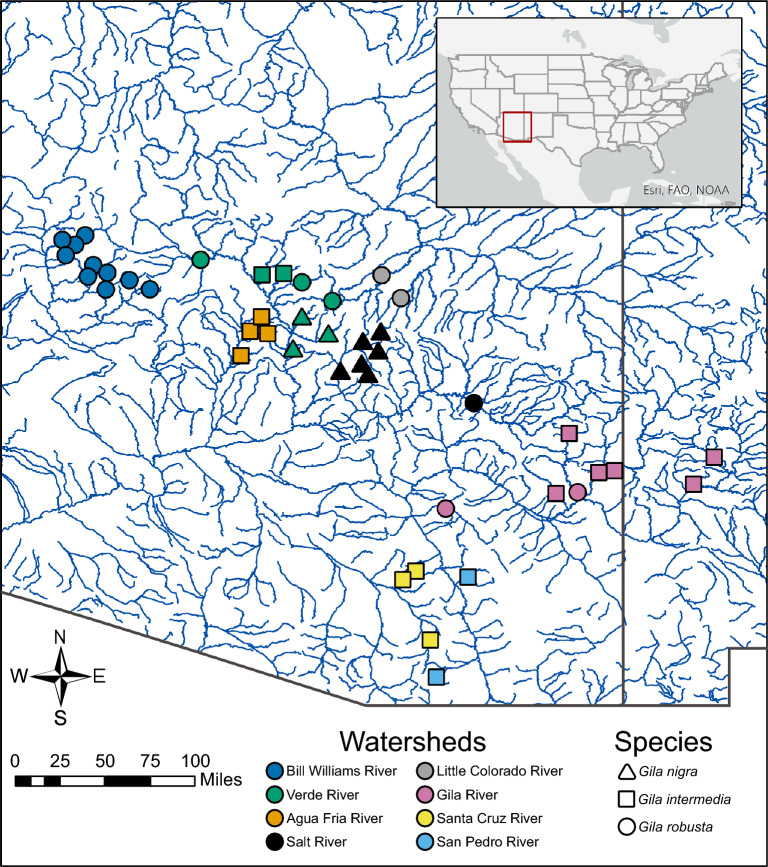
Sampling localities for *Gila robusta* species complex within the Lower Colorado River Basin, southwestern North America. Sampling points are colored by watershed with shapes indicating nominal species designation. The map was created in ArcGIS version 10.8.2 (https://www.esri.com) using open data sourced from ESRI (https://hub.arcgis.com/datasets/esri::usa-rivers-and-streams/explore).

Sampling locations and nominal species identifications for all 624 Next-RAD samples (*G. robusta* N = 266, *G. intermedia* N = 241, and *G. nigra* N = 117) are presented in Fig. 1. The draft *Gila robusta* genome and all raw sequence data were submitted to NCBI where they are made publicly available under BioProject number PRJNA922577 (*Gila robusta* species complex phylogenetics). We recovered a total of 120 Gb from our Sequel II reads with a slight AT bias at 39.4 ± 0.037% GT. Actual base frequencies were A = 0.30, C = 0.20, G = 0.20, T = 0.30 with a total contig length of 1,229,467,638 bp. Genome assembly data are reported in Table 1 and the project has been deposited at GenBank under the accession JAVALU000000000.

**Table 1:** Summary statistics for draft genome assembly of *Gila robusta* (#SAMN35560685).

Genome scaffold total	6841	1229.475 Mbp	Coverage 100%
Genome contig total	6912	1229.468 Mbp	Coverage 100%
Genome scaffold N50/N90	387 kbp	2149 kbp	
Genome contig N50/N90	400 kbp	2203 kbp	
Max. scaffold length	8.317 Mbp		
Number of scaffolds > 50 KB	3103		
% genome in scaffolds > 50 KB	95.57%		

Out of 694 total samples sent to SNPsaurus for individual nextRAD-genotyping, 65 individuals were not sequenced because of low quality or quantity of DNA, 4 were removed post-sequencing for quality filtering, and one for uncertainty of the code assignment back to species ID from a typo in the double-blinding process, resulting in a total of 624 fish samples analyzed here. From the total catalog of consensus sequences, indels, and SNP loci with a minor allele frequency below our minimum cutoff (MAF = 3%) were removed, leaving 10,246 loci for downstream analysis.

### Phylogenetic analyses

**Figure 2:**
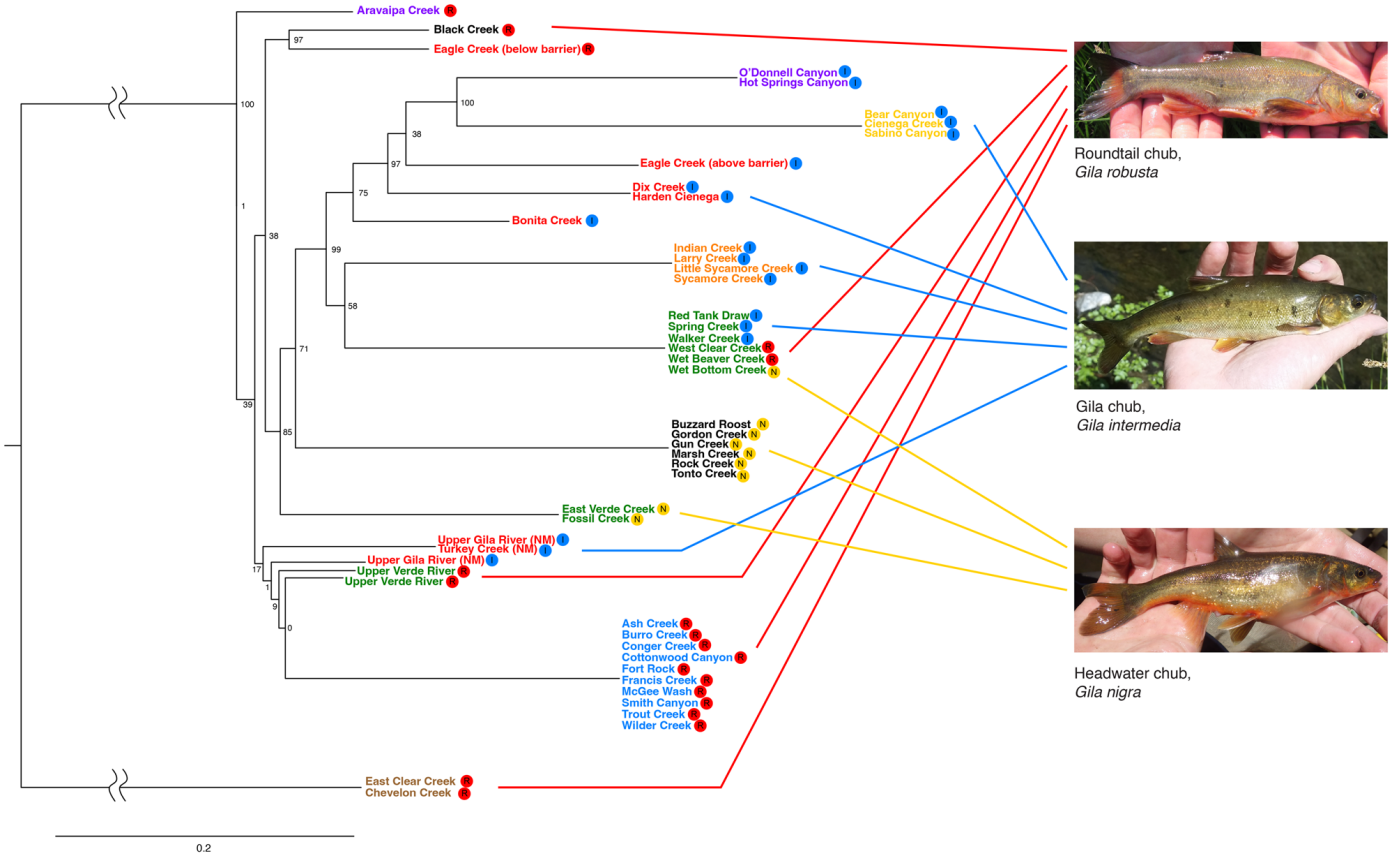
Phylogenetic tree of the *Gila robusta* complex. Site label color indicates the watershed for each stream location sampled: Little Colorado River (brown), Bill Williams River (blue), Verde River (green), Gila River (red), Salt River (black), Agua Fria River (orange), Santa Cruz River (yellow), San Pedro River (purple). Symbols following stream locations indicate taxonomic assignment to nominal taxa within the *Gila robusta* complex: *G. robusta* (red dot—R), *G. intermedia* (blue dot—I), *G. nigra* (mustard dot—N). Numerical values on the tree are maximum likelihood bootstrap support for each node. Photos courtesy of Arizona Game and Fish Department.

The first two splits in the phylogenetic tree generated by RAxML were well supported while most other nodes had considerably less support (Fig. 2). The Little Colorado River samples (Chevelon and East Clear Creeks) formed a highly divergent monophyletic group distinct from the rest of the samples, and the samples from Aravaipa Creek in the San Pedro Basin were divergent from all other non-Lower Colorado River samples. Despite large genetic distances among many regions, short internal branches often had little to no bootstrap support. Across sampling locations, all three of the *Gila robusta* complex nominal species are polyphyletic, whereas five of the eight watersheds formed monophyletic groups (Fig. 2). The RAxML output tree with all 624 individuals is included in the Supplementary Materials.

### Analyses of molecular variance (AMOVA)

AMOVA partitioned 23.12% of genetic variance as being explained among nominal species, whereas 30.92% of variance was explained via watersheds (Table 2). Pairwise *F*_*st*_ was significant (p < 0.001) across all comparisons, with the Little Colorado River standing out as highly distinct from all other sampling locations (Table 3). *F*_*st*_ values were highest between the Little Colorado River and other sites, ranging from 0.79 to 0.88, whereas the Gila River showed the lowest values with the adjacent watersheds, ranging 0.16 to 0.30 (Table 3). Beyond those two watersheds, the pairwise *F*_*st*_ values were intermediate and roughly proportional to the degree of geographic separation among sites.

**Table 2:** Analysis of molecular variance (AMOVA) testing alternate hypotheses using watersheds or nominal species within the *Gila robusta* complex as the unit of comparisons.

Source of variation	Sum of squares	Variance components	Percentage variation	Source of variation	Sum of squares	Variance components	Percentage variation
Among *species* groups	164,564.57	192.80	23.12	Among *watershed* groups	267,642.45	249.56	30.92
Among streams within *species* groups	260,691.36	242.82	29.11	Among streams within *watershed* groups	157,613.48	159.15	19.72
Among individuals within streams	183,547.83	−39.42	−4.72	Among individuals within streams	183,547.83	−39.42	−4.88
Within individuals	243,505.00	437.71	52.48	Within individuals	243,505.00	437.71	54.23

Note that the percentage of variation explained by the among streams component is highest in both scenarios, with watersheds explaining the majority of the genetic variance overall.

**Table 3:**
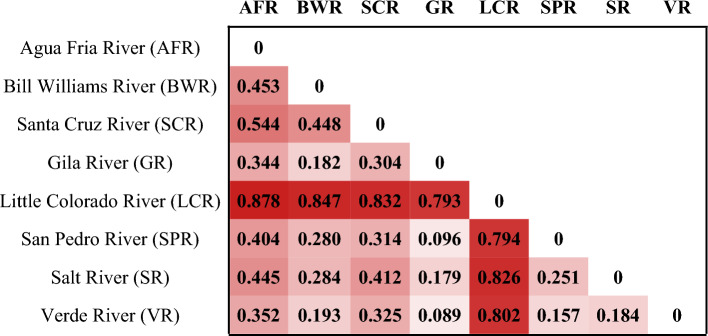
Pairwise *F*_*st*_ values between watersheds within the *Gila robusta* complex, with shading proportional to the magnitude of pairwise differences for ease of visualization.

All pairwise differences are significant after false discovery rate correction for multiple comparisons (P < 0.001).

### Structure analyses

**Figure 3:**
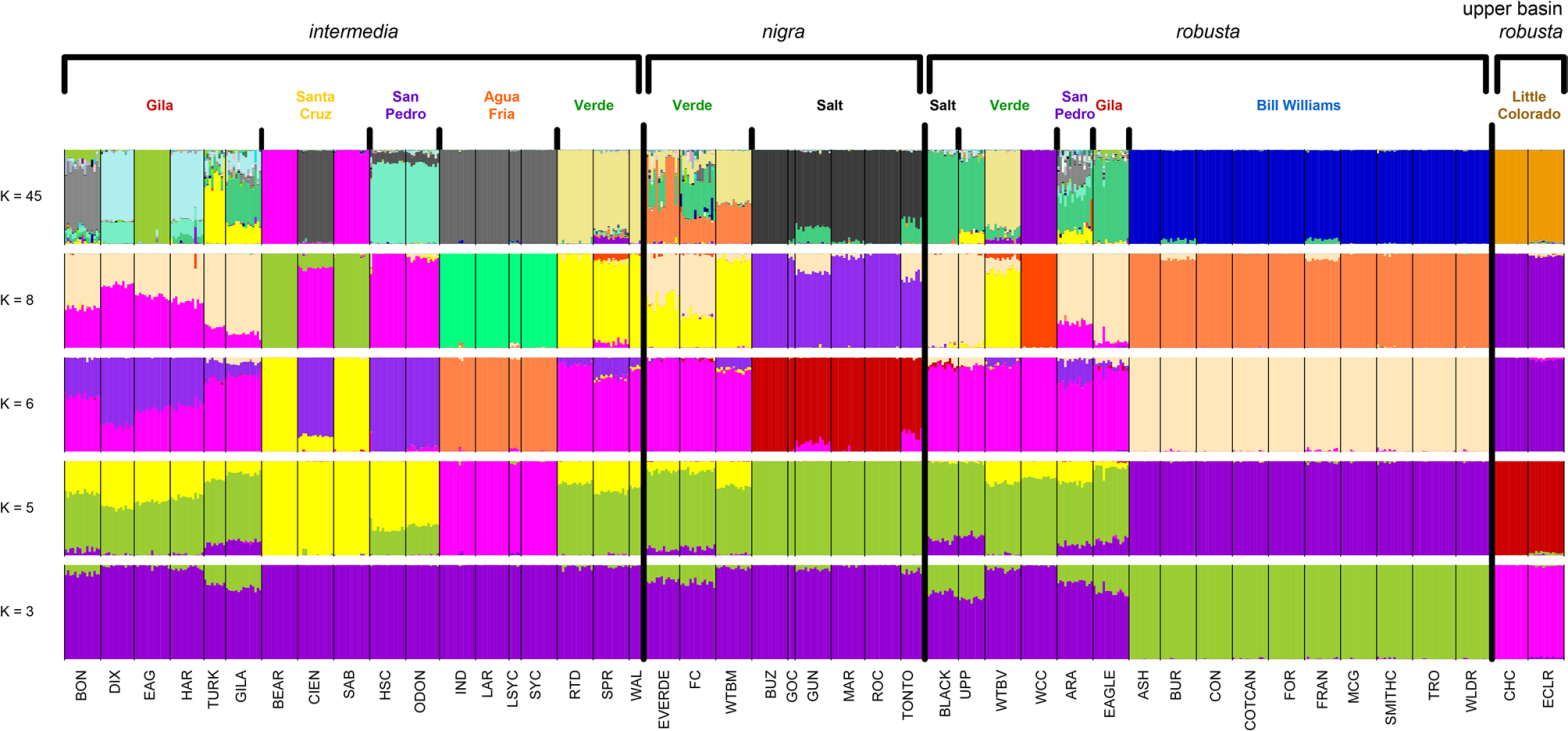
Assignment probability plots for all sample locations within the *Gila robusta* complex, including selected values of K based on a priori hypotheses of three nominal species (K = 3), uniquely identifiable streams (K = 45), and the ΔK criterion of K = 6 (Fig. 4). K = 5 and K = 8 are included as the most visually distinct patterns around the K = 6 optimum for comparison.

**Figure 4:**
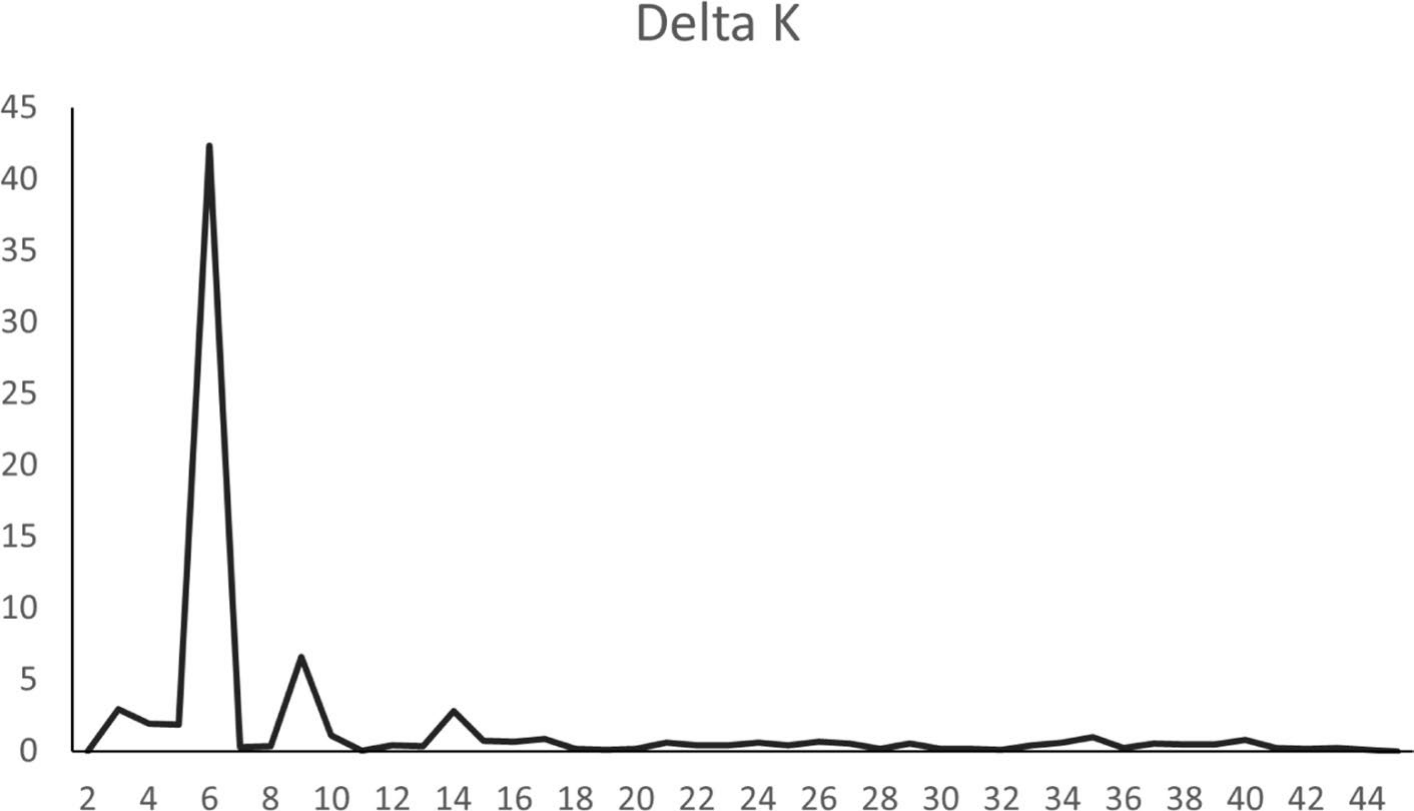
Delta-K (ΔK = mean (**|**L′′ (K)**|**/sd(L(K)) values plotted for the structure analysis of 2 to 45 groups within the *Gila robusta* complex, with K = 6 being the optimal value.

Structure plots for all values of K from 2 to 45 (the number of streams sampled in this study) were run to define populations and assign individuals back to them. A representative range of groupings (K values) is presented in Fig. 3. K = 3 was used to test the hypothesis that nominal species provide the best assignment of individuals. K = 6 provided the best fit based on the ΔK criterion, with K = 5 and K = 8 as the nearest peaks around K = 6 (Fig. 4). Finally, K = 45 is included as the hypothesis that each stream is a distinct genetic entity and would allow assignment back to the sampling location. In all cases, the *G. robusta* from the upper basin of the Little Colorado River are separated as a unique group, as do the samples from the Bill Williams River (BWR), but other locations are less consistent among groupings. Guided by the ΔK criterion, K = 6 shows high assignment of individuals from the Little Colorado River and BWR again, but also for the Salt River (SR), Agua Fria River (AFR) and San Pedro River (SPR) with partial assignments or apparent admixture of varying proportions among the remaining sampling locations.

### Discriminant analyses of principal components (DAPC)

**Figure 5:**
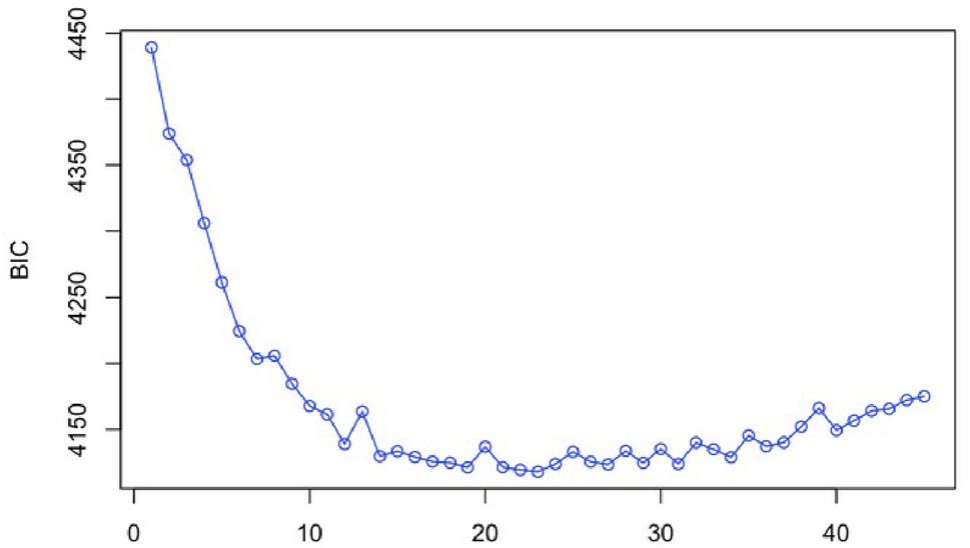
Bayesian information criterion (BIC) values for *k*-means clustering with k ranging from 2 to 45 groups within the *Gila robusta* complex, with K = 12 being the optimal value.

Twelve de novo genetic clusters were identified in our dataset using k-means clustering (Fig. 5). These clusters each contained 15–109 individuals from 1 to 8 streams in 1–4 watersheds (Supplementary Materials). Three of these clusters contained individuals from different nominal species based on meristics and sampling locations, with one genetic cluster containing all three nominal species (Supplementary Materials). The remaining clusters contained a single nominal taxon, but also only a single sampling location. Where multiple nominal species are collected at the same site, in almost all cases they group with other specimens from that same watershed to the exclusion of the same nominal species from other watersheds. The most consistent result from the k-means clustering is that individuals were assigned with high confidence back to their stream of collection, irrespective of the species identification. The single exception to this trend is one individual from Hot Springs Canyon in the San Pedro River watershed did not group with the other specimens from this stream. That specimen was assigned to cluster 3 rather than grouping with the other 14 specimens in cluster 4 (Supplementary Materials). Thus, only 1 out of 624 total fish in our study did not assign with high confidence back to the collection stream, highlighting the geographic distinctiveness of individual watersheds in this region.

**Figure 6:**
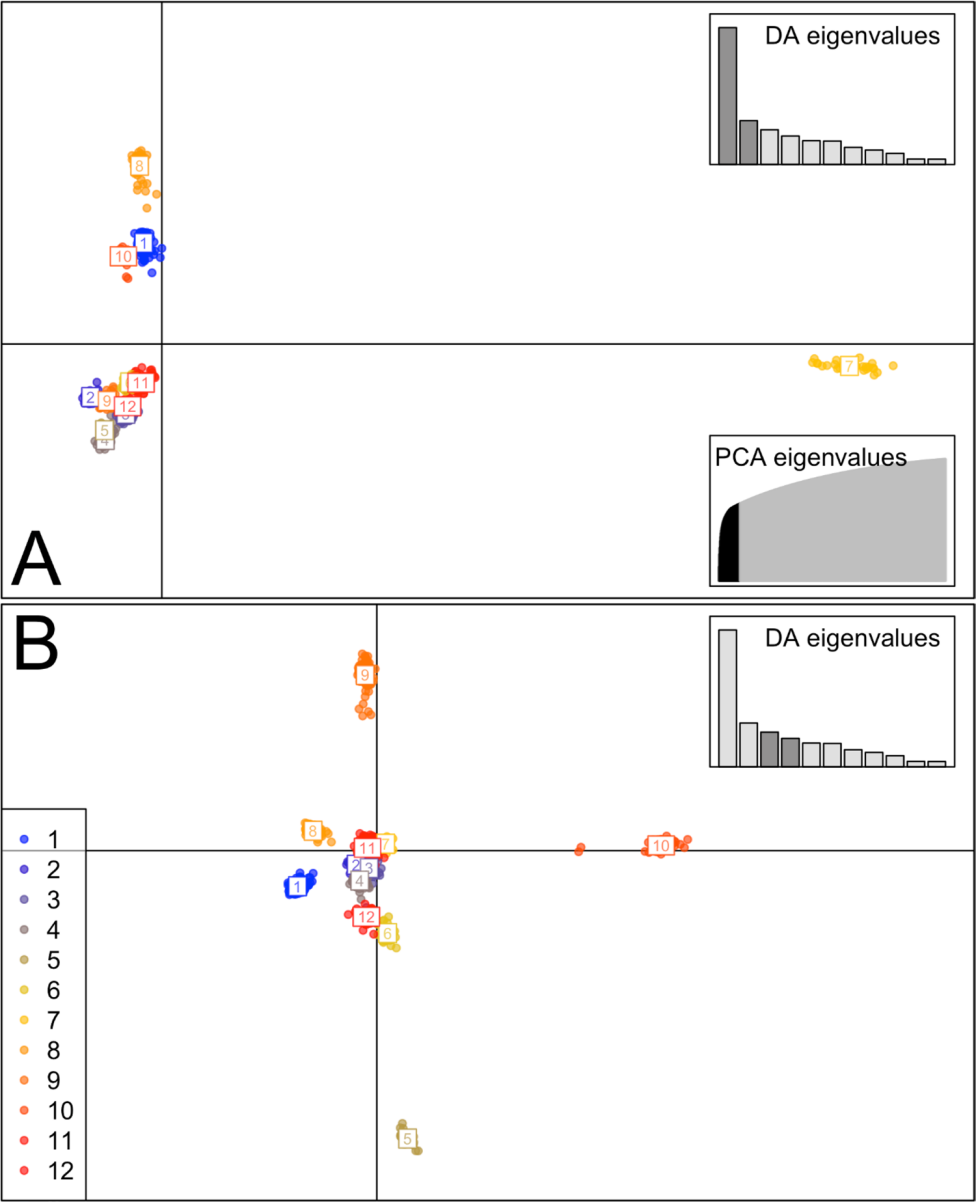
De novo clusters for the *Gila robusta* complex without a priori assumptions plotted on the first four discriminant functions (DFs) of the DAPC analysis. (**A**) The first and second discriminant functions: the x-axis shows DF1 and the y-axis shows DF2. (**B**) The third and fourth discriminant functions of the DAPC analysis: the x-axis shows DF3 and the y-axis shows DF4. The cumulative variance explained by the eigenvalues of the principal components analysis (PCA) and F-values for the discriminant analysis (DA) eigenvalues are inset.

Fifty-eight principal components were retained in the DAPC on the de novo clusters, and while all DFs were retained for further examination, only the first four are shown here (other DFs plotted in Supplementary Materials). The first DF (DF1) distinguishes samples from the Little Colorado River as highly distinct from all other samples (Fig. 6A). This cluster had 297 fixed nucleotide differences (loci fixed for one allele in the Little Colorado River/cluster 7 and fixed for the opposite variant in all other de novo clusters). The only other de novo cluster with any fixed nucleotide differences was cluster 10 (which had 6), and was the only other group consisting entirely of individuals from a single watershed (Agua Fria River). The second DF (DF2) distinguishes all samples from the two western-most watersheds (Bill Williams River represented in clusters 1 and 8, as well as the Agua Fria River in cluster 10; Fig. 6). DF2 therefore clusters different species within these drainages as more similar to one another than they are to putative conspecifics in other geographic locations, a result consistent with both the phylogenetic analyses and the structure assignments. The third DF distinguishes these two western watersheds from each other, while DF4 produces yet another geographic split in which multiple putative species are lumped together (Fig. 6B). For example, most samples from the Salt River watershed (cluster 9, all *G. nigra*) are split apart from those collected from the Verde River watershed that contains a mixture of species (cluster 5, *G. robusta*; cluster 6, *G. intermedia*; and cluster 12, ~ 2/3 *G. robusta* and ~ 1/3 *G. intermedia*).

**Figure 7:**
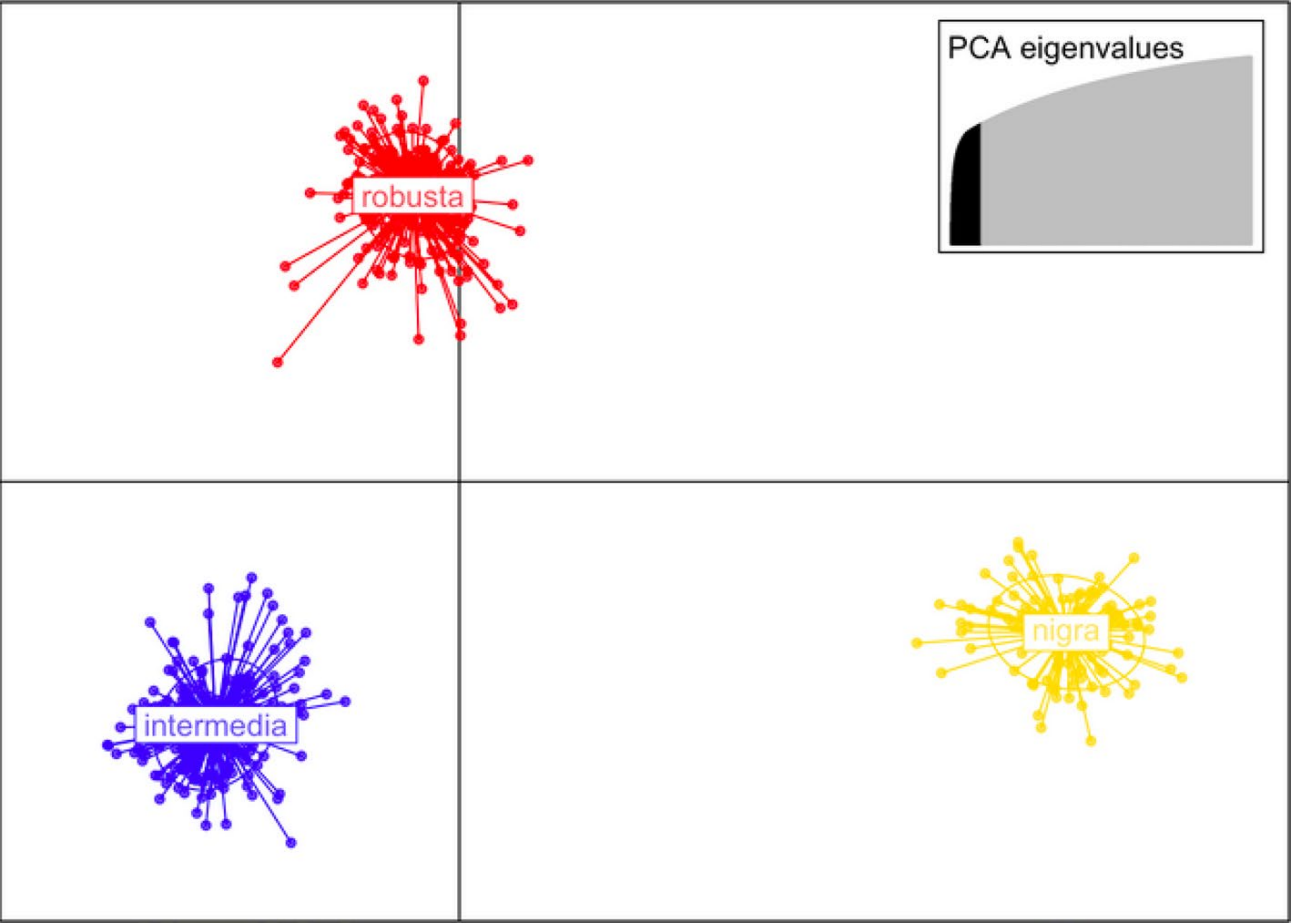
Forced clusters plotting of the *Gila robusta* complex on the first two discriminant functions (DFs) of the DAPC analysis with the a priori hypothesis that nominal taxa are valid. The x-axis shows DF1 and the y-axis shows DF2. Clustering based on the nominal taxa appear to support the distinction among species, however this pattern is not recovered unless names are used as priors, there are zero diagnostic SNPs among these clusters, and this clustering consistently explains less of the genetic variation than geography in any comparative analyses.

For comparison, we performed a DAPC analysis using the species names as priors and forcing the analysis to discriminate among nominal taxa based on these same data. Sixty-four principal components were retained in the DAPC and both DFs were plotted (Fig. 7). The three species were clearly distinguished when we analyze the data this way, but there are zero fixed nucleotide differences between any of the three putative species groups, and this DAPC explains less of the variation such that K = 3 was rejected by the BIC in the de novo analysis. Consistent with every other analysis presented herein, the data show that clustering by geography consistently explains more genetic variation than clustering by nominal species.

## Discussion

Freshwater habitats impose strong limits on the distribution of their resident biota. While dispersal in freshwater is contingent on several biological traits^32^, the combined roles of stream geomorphology, historical isolation and reticulation of habitats are major factors promoting freshwater biodiversity^2–5^. Species are expected to show some degree of dispersal within a given watershed, but gene flow should be far less common among watersheds. Except in cases of anthropogenic intervention, connections among distinct watersheds are based on either historical geomorphology or relatively rare flood events. As a result, the most common phylogenetic pattern observed in North American freshwater fishes are drainage-specific monophyletic lineages that cluster under a single species name^33–36^. The complex geological history of the southwestern United States makes the taxonomy of resident freshwater fish particularly challenging^37^. Overlaid on this general pattern, rapid evolution of freshwater fishes can produce differences in life history and morphology that further confound taxonomic resolution^9,11,38–40^. The nominal species of the *Gila robusta* complex are unique in that no other river basin in North America is known to contain a monophyletic group of species that cannot be distinguished by diagnostic morphological characters^23^. Despite considerable previous research, the taxonomy of the endemic Roundtail chub (*G. robusta*), Gila chub (*G. intermedia*), and Headwater chub (*G. nigra*) remain hotly debated.

Copus et al.^19^ provide a systematic and taxonomic review outlining the history of redescriptions, clerical errors, and confusion surrounding species within the genus *Gila*, with particular attention to the three nominal taxa *G. robusta*, *G. intermedia* and *G. nigra* that inhabit the lower reaches of the Colorado River. They found that both the types and fresh material exhibited as much or greater variation within as between nominal species, and that no character could be uniformly assigned back to the type specimens of *G. robusta*. Based on morphological characters, only 51% of *G. intermedia*, 63% of *G. nigra*, and 28% of *G. robusta* could be correctly aligned to the name-bearing type specimens. Subsequently, authors used both whole mitogenome and a reduced representation genome sequencing approach (with 89,896 loci) applied to a suite of phylogenetic approaches to evaluate genomic support for the three nominal species across the geographic range. Comparing 6 individuals of *G. robusta*, 6 of *G. intermedia*, and 5 of *G. nigra*, rooted with *G. elegans* and *G. cypha* (19 individuals), they found none of the three species formed a monophyletic clade; instead, all three nominal species were distributed among 5 clades throughout the phylogenetic reconstruction in Copus et al.^19^. Meanwhile, the American Society of Ichthyologists and Herptologists—American Fisheries Society (ASIH-AFS) Committee on the names of fishes found no evidence that *G. intermedia* and *G. nigra* were taxonomically distinct from *G. robusta*. Based on the absence of discrete morphological or genetic characters that could unambiguously identify the nominal taxa, both Page et al.^23^ and Copus et al.^19^ argued that this group formed a single polytypic species and that the rules of the International Commission of Zoological Nomenclature mandated synonymizing *G. intermedia* and *G. nigra* with *G. robusta*, which had priority.

Chafin et al.^25^ also review the contentious history of taxonomic status of the *Gila* species endemic to the lower Colorado River basin yet reach a different conclusion. They conducted the most extensive geographic and genomic sampling of fishes across this region to date with 386 individuals scored for 7,357 to 21,007 SNPs depending on the filtering thresholds for the data. They focused specifically on the phylogenetic conflict among previous studies and used SNP-based coalescent to test the hypotheses of a single polytypic species versus distinct evolutionary lineages for *G. robusta*, *G. intermedia* and *G. nigra*. Historical reconstructions led Chafin et al.^25^ to conclude that rapid Plio-Pleistocene drainage evolution, with subsequent divergence within the “anomaly zone” of tree space (i.e., incomplete lineage sorting dominated by anomalous gene trees^31^) produced inconsistent gene trees with ambiguities that confounded prior studies. Authors tested, and rejected, hybridization as a possible explanation for the phylogenetic discord among previous studies. Based on dense spatial and genomic sampling with coalescent and polymorphism-aware phylogenetic models, they support all three species as evolutionarily independent lineages. However, their reconstructions of effective population sizes for *G. robusta*, *G. intermedia* and *G. nigra* as well as the divergence times of the taxa are not significantly different from one another, and the models support 3 rather than 5 divergence events among 6 putative congeners (*Gila elegans*, *G. seminuda*, *G. jordani*, *G. robusta*, *G. intermedia* and *G. nigra*).

The resolution of this conflict required a nearly complete genetic atlas across the species range, including a detailed population genomic survey of the *Gila robusta* complex. Here, we report the results of this range-wide survey with the first draft genome for the species (SAMN35560685) and a reduced representation genomic approach to score 10,246 SNPs in each of 624 individuals sampled from throughout the lower reaches of the Colorado River (BioProject # PRJNA922577). We use these data to show that we reach the same conclusion as Chafin et al.^25^ about the number of distinct genetic groups present, that we can also support the three nominal species by post-hoc parsing of the data, and that nominal taxonomy never explains more of the variation than does geography in direct comparisons. Thus, we document how previous studies with both extensive geographic and genomic sampling reached conflicting conclusions about the taxonomic status of this group. We conclude that the reliance on locality as a taxonomic character confounds geographic structure with taxonomic resolution and conflates differences among the 3 nominal species when the divergence among watersheds exceeds the divergence among putative taxa. When collection site is used as a taxonomic character (certainly justified to minimize risk of mis-assigning species names) the geographic differentiation between drainages *becomes* the genetic distinction between nominal taxa, as highlighted by our multiple hierarchical analyses on double-blind samples.

Consistent with previous studies, the Little Colorado River *G. robusta* specimens are by far the most distinct, with 297 fixed nucleotide differences from the remaining samples throughout the range. The discriminant analysis of principle components (DAPC) shows that the second clearest genetic distinction (DF2) separates samples from the Bill Williams River watershed (*G. robusta*) and the Agua Fria River watershed (*G. intermedia*) from all other samples, while the third clearest genetic distinction (DF3) separates the samples from these two watersheds from each other. The fourth clearest genetic distinction (DF4)—out of the eleven mapped in the DAPC analysis—produces yet another geographic split which contains multiple nominal species grouped together and makes clear labeling of the cluster membership impossible (interested readers will find additional plots, code and all the details of the analyses in the Supplementary Materials). Most of the Salt River watershed (*G. nigra*) are split apart from the Verde River watershed (represented by one group of putative *G. robusta*, one group of putative *G. intermedia*, and a third group that combines putative *G. robusta* and *G. intermedia*). Notably the clusters defined by DAPC do not imply that each watershed contains a well-mixed population. For example, in the Salt River drainage, all nominal *G. intermedia* form a single monophyletic clade that does not include the two putative *G. robusta* from the same watershed. However, those two sites (CHER and BLACK) are geographically separated from the remainder of the Salt River watershed, so these findings could be attributed to geographic partitioning as easily as they could to taxonomy. Likewise, where *G. robusta*, *G. nigra* and *G. intermedia* were sampled from the various regions of the Verde River drainage, they group together in the same clade rather than matching the nominal taxonomic labels from other locations. Following the practice of defining species by their sampling locations, we also performed a DAPC procedure as above but with a priori categorization into the three putative species. When species identity is used to define groups for the analyses, we clearly find support for these groupings, but this is confounded with geographic sampling location, which is the primary explanatory variable from all the unconstrained analyses. For comparison, AMOVA partitions 23% of the genetic variation among the nominal taxa, but watershed explains nearly 31% of the variance, irrespective of species ID (Table 2). In each of these analyses, species integrity can be maintained if the geographic context is taken as a reliable taxonomic character, but the fact remains that we see greater structure by watersheds than by nominal taxonomy in every analysis here. Thus, using a blinded sampling design and analyses confirm that in every case in which nominal taxonomy can be compared directly with geographic partitioning of the same genomic data, geographic destiny explains more of the observed variation.

As Chafin et al.^25^ observed, when speciation events are rapid and population sizes are large, there may not be sufficient time to sort ancestral variation in the populations, such that the most probable gene topologies can conflict with the underlying species divergence (incomplete lineage sorting^31,41,42^). This results in what has been coined an “anomaly zone” of tree space. Inferring species trees is demonstrably difficult in this region^43^, and exceedingly so if additional sources of phylogenetic discordances, such as translocations, reintroductions, or hybridization are also occurring^44^. We fully acknowledge the issues of large population size and rapid diversification outlined by Chafin et al.^25^ regarding the anomaly zone where many of the most challenging taxa reside. However, such strikingly divergent conclusions reached by previous researchers using genomic scale data with many thousands of loci each^19,25^ is hard to reconcile and important to understand, both for future management in this system, but also for the reliability of conservation genomics applied to natural systems. Here we undertake an intentional survey of as much of the geographical and taxonomic variation as could be obtained, using blinded samples being analyzed before comparing geographic and taxonomic hypotheses using a suite of hierarchical analyses to explain why and how previous studies can come to such conflicting conclusions. The simple answer to the conflict appears to be a priori concerns about taxonomic identification conflating geographic structuring among watersheds, complicated by frequent changes to Plio-Pleistocene drainages, a common conclusion for freshwater fishes^3–5,45^. In this situation, researcher concerns about taxonomic uncertainty would result in sampling bias to obtain “pure stocks” that would reinforce preconceived notions about species identity.

For example, our findings include clear phylogenetic indications that some watersheds host a single clade of what was previously identified as one of the three nominal species. Clearly some drainages, such as the Little Colorado, Bill Williams, and Agua Fria Rivers, emerge as distinct from the remainder of the range. Likewise, while there is sufficient signal to support three nominal taxa, any sampling of type localities to ensure “pure” stock of the nominal taxa, or assignment of taxonomy based on sampling location, will always support individuals from different localities being distinct because of the geographic structuring of watersheds. However, if we sample across the geographic range of these fish and compare genomic signatures of population structure, every analysis consistently finds the nominal taxa within a watershed are more similar to one another than they are to the same species in other watersheds (e.g., Dowling et al.^46^). Structure detects 6 groups to which individuals can be assigned with high confidence, whereas k-means clustering through DAPC with BIC identifies 12 genetically distinct groups, most similar to the phylogenetic clades resolved with high bootstrap values. Likewise, Chafin et al.^25^ found K = 11 as the optimal solution for their genomic sampling, with significant structure at the drainage or sub-drainage level. None of the analyses herein or published previously identify 3 groups as the best fit to the data, and here we show that when species identity is used as a prior, the proportion of the variation explained always decreases relative to comparisons by watershed. As Chafin et al.^25^ point out, “if a priori taxon assignments are evolutionarily independent, then they should be recapitulated in the phylogeny, irrespective of the drainage partition from which populations were sampled.” In contrast, a single polytypic species should show that more of the variation is explained by stream hierarchical structuring. Our analyses show the latter is true, indicating that genetic structure reflects intraspecific processes rather than evolutionarily independent lineages within the *G. robusta* complex.

### Management implications

Notably, the Lower Colorado River is somewhat of an artificial construct, being separated from the Upper Colorado River by the Glen Canyon Dam, which was completed in 1964. Since that time, many freshwater fishes in the lower basin have declined, due to water quality changes and habitat alteration that put them in direct conflict with the water needs of a growing economy and community in the Southwest U.S.A.^47^. In 2022, drought brought the corresponding reservoir (Lake Powell) to the lowest level since construction, so that management of scarce water exerts tremendous pressure on other resources, including aquatic wildlife. All these factors lend greater urgency to conservation measures aimed at the *Gila robusta* species complex and other endemic species of the lower reaches of the Colorado River.

The taxonomic issue of whether these species are valid stands in parallel to the question of whether *Gila* spp. in the lower reaches of the Colorado River need protection and conservation measures. The Endangered Species Act defines a species to include “any subspecies of fish or wildlife or plants, and any distinct population segment of any species of vertebrate fish and wildlife which interbreeds when mature” (Section 3 (15), ESA 1973, 1978). However, this conflict stems from the fact that *G. intermedia* and *G. nigra* are defined as distinct species based on mean differences in meristic counts between populations inhabiting different streams^21,48,49^, and none of the studies to date indicate a single well-mixed population across the Lower Colorado River Basin. Thus, if there is any uncertainty in species identification, the default for researchers who consider the species as valid entities has been to use the locality for species identifications^19^. If one has an a priori expectation that the nominal species are valid, and sample taxonomy is based on locality, then analyzing the data based on those groupings is clearly supported (Fig. 7) but also confounded by geographic population structuring (Table 2). Here we confirm the finding of Chafin et al.^25^ that there are 10–12 distinct genetic groups among these watersheds but contest the conclusion that those groups comprise three valid species. We show how extensive studies with genomic scale data can reach conflicting conclusions and resolve the conflict between previous studies by showing both can be supported with our data set based on the inclusion or exclusion of samples from the analyses^19,25^. The loss of any of these watershed populations would cause a disproportionate reduction in genetic diversity which potentially translates to reduced fitness and increased risk of extinction, principles that apply to a wide range of species^50–54^. Thus, we recommend that *G. robusta* be managed on the basis of watersheds as the primary unit of divergence, rather than on nominal taxonomy.

Overall, each analysis we present here confirms that geography explains more of the variation than does nominal taxonomy. In addition, while there are fixed SNP differences among watersheds, there are zero fixed differences between the nominal species within the *Gila robusta* complex. This finding builds on the analyses of Copus et al.^19^ and Carter et al.^20^ who showed that no diagnostic characters exist for morphology either. Because morphological and genetic distinctiveness covary in this system, there are morphometric analysis that can discriminate among the nominal taxa^20–23^, just as we can force support for the nominal species post-hoc in our analyses (Fig. 7), but direct tests of taxonomy versus geography for explaining the variation always favor geographic population structuring. Likewise, because ICZN code requires that there be at least one diagnostic character upon which a species can be unambiguously assigned to a type, the absence of any single morphological or genetic character that could assign a fish to one of the three nominal species would preclude their recognition as valid species under the code today. Our study reconciles apparently discordant previous work and reinforces the determination of the AFS/ASIH Joint Committee on the names of fishes that *Gila robusta* should be recognized as a single [polytypic] unit as per Page et al.^23,27^.

## Conclusions

We show that a sample strategy based on taxonomic expectations results in unintentional biases toward supporting preconceived notions that the nominal species are valid. Indeed, there is support for both geographic and taxonomic partitions depending on how the study is designed, how the data are parsed, and which analyses are used. In an evolutionary lineage such as *Gila robusta* with strong population genetic structuring, species definitions become circular if taxonomy is defined by sample location. Authors who struggle with taxonomic uncertainties are likely to focus on type localities to prevent misidentifications, an approach which would bias their results *toward* supporting the nominal species. In contrast, a sampling design to capture the breadth of genetic variation across the species range could potentially bias results *away* from supporting the three nominal species. Both approaches are understandable and scientifically justifiable in isolation but come into conflict when each supports a different conclusion^19,25^ with direct implications for management actions.

Evolutionary lineages within *Gila robusta* are largely defined by watersheds irrespective of the taxonomy applied (both data herein and Chafin et al.^25^). Why does such strong support for recognizing *G. intermedia* and *G. nigra* persist? In recent decades, there has been a tendency for conservationists to accept dubious taxonomy for the purpose of protecting wildlife within existing legal frameworks^55^. There are scientifically sound reasons for conservation of *Gila robusta*, but spurious taxonomy should not be one of them. Conservation priorities will change over time to allow for adaptive management, but taxonomy should be shaped by scientific data as applied through the rules of the International Commission of Zoological Nomenclature. In the absence of diagnostic molecular or morphological characters between taxa within the *Gila robusta* complex, and greater morphological dissimilarity among the name-bearing types than between sister taxa^19^, the conclusion based on ICZN criteria is clearly a single polytypic species. While these findings may simplify the taxonomy of *Gila* spp., they also confound management of *Gila robusta*. With strong isolation of watersheds (*F*_ST_ = 0.31) and weaker (but significant) isolation of streams within watersheds (*F*_ST_ = 0.19), it seems clear that the watersheds are distinct populations and should be managed as such. Regardless, management and conservation of these fishes should concentrate on maintaining genetic diversity and morphological variation present among watersheds, rather than three nominal species for which there is variable and inconsistent support.

## Materials and methods

### Field collection & DNA extractions

Currently there is no reliable method to unambiguously identify the three species of the *Gila robusta* complex morphologically in the field^20,22,23^. Species assignments by wildlife managers are currently based on drainage location as originally assigned in Rinne^48^ and later revised by Minckley and DeMarais^21^. Lacking alternative methods of species identification, we follow these location-based species assignments in accordance with the literature. Fresh specimens of each nominal species were obtained by trained members of Arizona’s Game and Fish Department from 48 stream sites residing within 8 watersheds, with additional specimens from Eagle Creek, East Clear Creek, and East Verde River provided by the Bubbling Ponds Fish Hatchery for a total of 694 samples (Fig. 1). Multiple sampling sites across a broad geographic range were chosen to capture as much of the species range as possible. All specimens were collected and provided by the State of Arizona Game and Fish Department, and all methods were carried out in accordance with relevant state and federal guidelines and regulations. All methods followed ARRIVE guidelines, and samples were processed following experimental protocols approved by the University of Hawai‘i Institution Animal Care and Use Committee (IACUC) protocol (#15-2271-3) to B.W.B.

Fin clip tissue samples were stored in 95% EtOH prior to DNA extraction. Genomic DNA was extracted from fin clip tissue using the Omega E.Z.N.A Tissue DNA Kit (Omega Biotek, Norcross, GA, USA) following the manufacturer’s protocol with an addition of 5uL RNase A. Extracted DNA was visualized by electrophoresis on a 1% agarose gel to assess quality and quantified using an Invitrogen Qubit Flex Fluorometer (Thermo-Fisher Scientific, Foster City, CA, USA). All extractions were stored at −20 °C prior to shipping to SNPsaurus LLC (Eugene, OR, USA) for independent processing.

### *Gila robusta* genome, nextRAD Sequencing, and SNP calling

To ensure that the differing RAD approaches selected by Copus et al.^19^ and Chafin et al.^25^ did not underlie the divergent conclusions, we selected an independent lab to perform the genetic analyses for this study. One *Gila robusta* sample (WCC17-021) was prepared for PacBio long-read sequencing carried out at SNPsaurus LLC (Eugene, OR). The sequencing library was prepared using the SMRTbell Express template preparation kit v2.0 (Pacific Biosciences, Menlo Park, CA) according to the manufacturer’s protocol. The sequencing library was size selected using with the BluePippin system (Sage Science, Beverly, MA) with a 0.75% DF Marker S1 high-pass 6- to 10-kb v3 cassette (Sage Science) according to the manufacturer’s recommendations. A size-selection cutoff value of 8000 bp (BP start value) was used. The size-selected SMRTbell library was annealed and bound according to the SMRT Link setup (Pacific Biosciences) and was sequenced on a Sequel II system in portions of two SMRT cells. The combined de-multiplexed bam files were converted to fasta format with SAMtools^56^ and used as input for Flye 2.7-b1585^57^ with an estimated genome size of 1.6 Gb, using parameters: flye --pacbio-raw pac33.RT_021.fa pac34.RT_021.fa --genome-size 1600 m --out-dir RT_021combined --threads 88. Contigs were tested for bacteria, fungi or other possible contaminants using blastn, but none were found. Thus, the full assembly.fasta file was annotated with augustus v.3.3.3^58^ using zebrafish as a model, with parameters: augustus --gff3 = on --species = zebrafish RT_21_Gila_refv1.fa. The predicted proteins were extracted and run with blastp versus zebrafish predicted proteins using the NCBI *Danio rerio* protein set. The blastp results were then added back to the gff file. All raw sequence data is publicly available under BioProject #PRJNA922577.

All individual DNA extractions from this study were coded such that samples could be run blindly without knowledge of the species ID or site of origin through nextRAD (Nextera-tagmented, reductively-amplified DNA) genotyping-by-sequencing to collect SNP data^59^. This nextRAD approach uses selective PCR primers to amplify genomic loci consistently between samples. Genomic DNA was first fragmented with Nextera reagent (Illumina, Inc, San Diego, CA, USA), which also ligates short adapter sequences to the ends of the fragments as outlined in Russello et al.^60^. The Nextera reaction was scaled for fragmenting 15 ng of genomic DNA, although 60 ng of genomic DNA was used for input to compensate for degraded DNA in the samples and to increase fragment sizes. Fragmented DNA was then amplified for 27 cycles at 74 °C, with one of the primers matching the adapter and extending ten nucleotides into the genomic DNA with the selective sequence GTGTAGAGCC. Thus, only fragments starting with a sequence that can be hybridized by the selective sequence of the primer will be efficiently amplified. The resulting fragments are fixed at the selective end and have random lengths depending on the initial Nextera fragmentation. Because of this, amplified DNA from a particular locus is present at many different sizes and careful size selection of the library is not needed prior to sequencing. These nextRAD libraries were sequenced on a HiSeq 4000 with four lanes of 150 bp reads at the University of Oregon (Eugene, OR, USA). HiSeq reads were then mapped to the draft *Gila robusta* genome and SNPs called as in Russello et al.^60^. The genotyping analysis used custom scripts developed by SNPsaurus LLC that trimmed the reads using bbduk (BBMap tools, http://sourceforge.net/projects/bbmap/). Mapping to the reference genome included an alignment identity threshold of 0.95 using bbmap (BBMap tools). Genotype calling was done using *callvariants* (BBMap tools). The resulting vcf was filtered using VCFtools^61^ to remove alleles with a population frequency of less than 3% (MAF) and individual samples with more than 50% missing data. We performed an initial analysis with 10 to 50% missing data and confirmed that the threshold did not result in a qualitative change in the results, so we opt for the most permissive threshold of missing data to include as much data as possible here. Only after SNP calling was each code reassigned to a collection location and species ID for the final analyses.

### Phylogenetic analyses

Trees were created via maximum likelihood (ML) analyses using the randomized accelerated maximum likelihood next generation (RAxML-NG) software v.1.0.0^62^ with the GTR + ASC_LEWIS + G evolutionary model. RAxML-NG tests for model convergence every 50 bootstraps and stopped after 1300 replicates with these data. Phylogenetic trees were constructed and visualized using FigTree v.1.4.2 (http://tree.bio.ed.ac.uk/software/figtree/).

### Analyses of molecular variance (AMOVA)

Arlequin 3.5^63^ was used to test whether watershed or nominal species assignment explains more of the variation in the data. In the first AMOVA, populations were assigned to one of three nominal species groups (*G. robusta*, *G. intermedia*, or *G. nigra*) to quantify how much variation is explained by taxonomy. In a separate analysis, samples were assigned to one of eight watersheds (Gila River, Verde River, San Pedro River, Salt River, Santa Cruz River, Little Colorado River, Agua Fria River, Bill Williams River), irrespective of taxonomic identity, to determine how much of the genetic variation is explained by geography. Arlequin 3.5 was also used to calculate pairwise *F*_*st*_ values between each sampling site.

### Structure analyses

Patterns of population structure were visualized using STRUCTURE^64^ implemented via the ParallelStructure^65^ package in R^66^. Models with a priori groups (K) ranging from 2 to 45 were evaluated, each with 20 independent replicates of 160,000 iterations (burn-in = 10,000) performed. Exclusion of the most divergent populations (Little Colorado and Agua Fria Rivers which contain private alleles) did not alter the conclusions, so all populations are included as the a priori design. The optimal number of groups (K) was determined using the method of Evanno et al.^67^ as implemented in STRUCTURE HARVESTER^68^.

### Discriminant analyses of principal components (DAPC)

Discriminant analysis (DA) maximizes the separation between groups while minimizing variation within each group, providing superior power in multidimensional space^69^. Discriminant Analysis of Principal Components (DAPC) is a powerful and assumption free tool to identify population partitions based on the large volume of data available from genomic studies^69^. DAPC analyses were carried out using the adegenet^70^ R package. After importing the data into R using the vcfR package^71^, two DAPCs were performed. The first used de novo groups generated by *k*-means clustering to determine the optimal number of genetic clusters (K) between 2 and 45. Multiple selection criteria based on Bayesian information criterion (BIC) in the find.clusters() function in adegenet yielded the same optimal *k*-value, so we used the *k* clusters selected by the “goodfit” criterion. The membership of each cluster was recorded at the stream, watershed, and species level. The number of clusters contained in each watershed and species group was also recorded. The second analysis used a priori groups (the three putative species). We used a-score (the optim.a.score() function in adegenet) to determine the optimal number of principal components to retain in both DAPC analyses. All samples were plotted along the main discriminant functions (DFs) and examined visually. Details of the analyses, code, and exclusion of the most divergent populations are included in Supplementary Materials. DAPC methods and results are reported according to the recommended standards in Miller et al.^72^. The level of differentiation between both the de novo (geographic) and a priori (taxonomic) groupings was also quantified by tallying the number of fixed nucleotide differences within each group, using the dplyr R package^73^.

### Supplementary Information

Supplementary Information.

## Data Availability

The datasets generated and analyzed during the current study are available in the National Center for Biotechnology Information (NCBI) repository, and are publicly available under BioProject Accession Number PRJNA922577. The draft *Gila robusta* whole genome shotgun project has been deposited at DDBJ/ENA/GenBank under the Accession Number JAVALU000000000. The version described in this paper is version JAVALU010000000. The R markdown for our analyses is included as Supplementary Materials.
