# Supplementary material for: Geographic destiny trumps taxonomy in the Roundtail Chub, Gila robusta species complex (Teleostei, Leuciscidae)
Source: Sci Rep. 2023 Sep 22;13:15810. doi: 10.1038/s41598-023-41719-9 (PMC10517014; doi:10.1038/s41598-023-41719-9)
Supplement: Supplementary file 1 — Supplementary Information. [file 41598_2023_41719_MOESM1_ESM.pdf]

Geographic destiny trumps taxonomy in the Roundtail Chub, *Gila robusta* species complex (Teleostei, Leuciscidae)

Suchocki, Ka'apu-Lyons, Copus, Walsh, Lee, Carter, Johnson, Etter, Forsman, Bowen & Toonen

Watershed	Species	Stream	Code	nSamples
**Bill Williams n=151**	robusta	Ash Creek	ASH	13
		Burro Creek	BUR	15
		Conger Creek	CON	15
		Cottonwood Canyon	COTCAN	15
		Fort Rock	FOR	15
		Francis Creek	FRAN	15
		McGee Wash	MCG	15
		Smith Canyon	SMITHC	15
		Trout Creek	TRO	18
		Wilder Creek	WLDR	15
**Verde River n=122**	robusta	Upper Verde River	UPP	11
		Wet Beaver Creek	WTBV	15
		West Clear Creek	WCC	15
	intermedia	Walker Creek	WAL	6
		Red Tank Draw	RTD	15
		Spring Creek	SPR	15
	nigra	East Verde River (BPH)	EVERDE	15
		Fossil Creek	FC	15
		Wet Bottom Creek	WTBM	15
**Salt n=86**	robusta	Black River	BLACK	14
	nigra	Buzzard Roost	BUZ	15
		Gordon Creek	GOC	3
		Gun Creek	GUN	15
		Marsh Creek	MAR	14
		Rock Creek	ROC	15
		Tonto Creek - Hell's Gate	TONTO	10
**Gila n=97**	robusta	Eagle Creek (Below Barrier)	EAGLE	15
	intermedia	Bonita Creek	BON	15
		Dix Creek	DIX	14
		Eagle Creek (Above Barrier)	EAG	15
		Harden Cienega	HAR	14
		Turkey Creek (NM)	TURK	9
		Upper Gila River (NM)	GILA	15
**Agua Fria n=49**	intermedia	Indian Creek	IND	15
		Larry Creek	LAR	14
		Little Sycamore Creek	LSYC	5
		Sycamore Creek	SYC	15
**Santa Cruz n=45**	intermedia	Bear Canyon	BEAR	15
		Cienega Creek	CIEN	15
		Sabino Canyon	SAB	15
**San Pedro n=44**	robusta	Aravaipa Creek	ARA	15
	intermedia	Hot Springs Canyon	HSC	15
		O'Donnell Canyon	ODON	14
**Little Colorado River n=30**	robusta	Chevelon Creek	CHC	15
		East Clear Creek (BPH)	ECLR	15

(BPH) = Fish acquired via Bubbling Ponds Fish Hatchery

Supplementary Table 1: Sampling locations by stream aggregated by watershed and nominal species designation for each. nSamples = the number of individuals sampled and Code = 3 letter shorthand used for the site throughout the manuscript. Note that Larry Creek is a self-sustaining introduced population with the donor population coming from Silver Creek, and Bear Canyon is a self-sustaining introduced population with the donor population coming from Sabino Canyon. Resource managers refer to these self-sustaining introductions as 'replicate populations' and replicated streams in this study include: Larry/Lousy creeks, Blue River, Ash Creek, Romero Canyon, Bear Canyon, Roundtree Canyon, Webber Creek, and Rarick Canyon. The East Verde River population is a remnant population, in which fish from there were held for a short time at the Bubbling Ponds Hatchery (which is where the sample was taken). The Upper Gila River sample came from Turkey Creek New Mexico and was provided by the Department of Game and Fish.

A central conclusion of this manuscript is that arbitrary exclusion of a subset of samples allows the data to support preconceived notions of the observer. We designed and ran all analyses for this study in double-blind fashion, which is the gold standard in biology for avoiding unintentional biases by researchers. We intentionally sampled the full geographical and genetic range of the distribution for these fish and found that the distribution of genetic variation in this system is such that researchers can very easily find support for their preconceived notions through post-hoc rationalizations about which samples to include in the analysis.

In the review process we were asked to remove the most divergent populations from the analysis to show that our conclusions were robust. The problem with post-hoc exclusion of samples in this system is that we can reconstruct support for divergent conclusions simply by excluding certain geographic samples from the study (Supp Fig. 1). We designed the study to sample the full range of geographic and taxonomic variation for which we could obtain samples within this group, and then generated the data and performed the analyses on coded samples so that everyone was blind to the site of origin and nominal taxonomy. Only after generating the data did we reassign the sample identifiers and explicitly test the alternative hypotheses of nominal taxonomy versus geographic structuring. Thus, we are opposed to any post-hoc manipulation of which samples are included in the analyses, because that decision can greatly bias the outcome of the analyses and conclusions drawn from them. For example, exclusion of a handful of sites for high levels of divergence or taxonomic uncertainty of the samples in that watershed can recover support for monophyletic lineages that support preconceived notions about the validity of the nominal taxa, but that are at odds with the underlying distribution (Supp Fig. 1), in the same way that using species names as priors in the DAPC can recover support for those names because morphological, genetic and geographic distinctiveness are confounded in this system.

Supplementary Figure 1: Phylogenetic tree of the *Gila robusta* complex based on 10,246 SNPs. Site label color indicates the watershed for each stream location sampled: Little Colorado River (brown), Bill Williams River (blue), Verde River (green),

Gila River (red), Salt River (black), Agua Fria River (orange), Santa Cruz River (yellow), San Pedro River (purple). Symbols following sample ID indicate taxonomic assignment to nominal taxa within the *Gila robusta* complex: *G. robusta* (red dot - R), *G. intermedia* (blue dot - I), *G. nigra* (mustard dot - N). Sample IDs indicate stream of origin: Aravaipa Creek (ARA), Ash Creek (ASH), Bear Canyon (BEAR), Black River (BLACK), Bonita Creek (BON), Burro Creek (BUR), Buzzard Roost (BUZ), Chevelon Creek (CHC), Cienega Creek (CIEN), Conger Creek (CON), Cottonwood Canyon (COTCAN), Dix Creek (DIX), Eagle Creek (EAG), Eagle Creek-below barrier (EAGLE), East Clear Creek (ECLR), East Verde River (EVERDE), Fort Rock (FOR), Fossil Creek (FC), Francis Creek (FRAN), Gordon Creek (GOC), Gun Creek (GUN), Harden Cienega (HAR), Hot Springs Canyon (HSC), Indian Creek (IND), Larry Creek (LAR), Little Sycamore Creek (LSYC), Marsh Creek (MAR), McGee Wash (MCG), O'Donnell Canyon (ODON), Red Tank Draw (RTD), Rock Canyon (ROC), Sabino Canyon (SAB), Smith Canyon (SMITHC), Spring Creek (SPR), Sycamore Creek (SYC), Tonto Creek (TONTO), Trout Creek (TRO), Turkey Creek (TURK), Upper Gila River (GILA), Upper Verde River (UPP), Walker Creek (WAL), Wet Beaver Creek (WTBV), Wet Bottom Creek (WTBM), Wilder Creek (WLDR), West Clear Creek (WCC). Numerical values on the tree are maximum likelihood bootstrap support for each node.

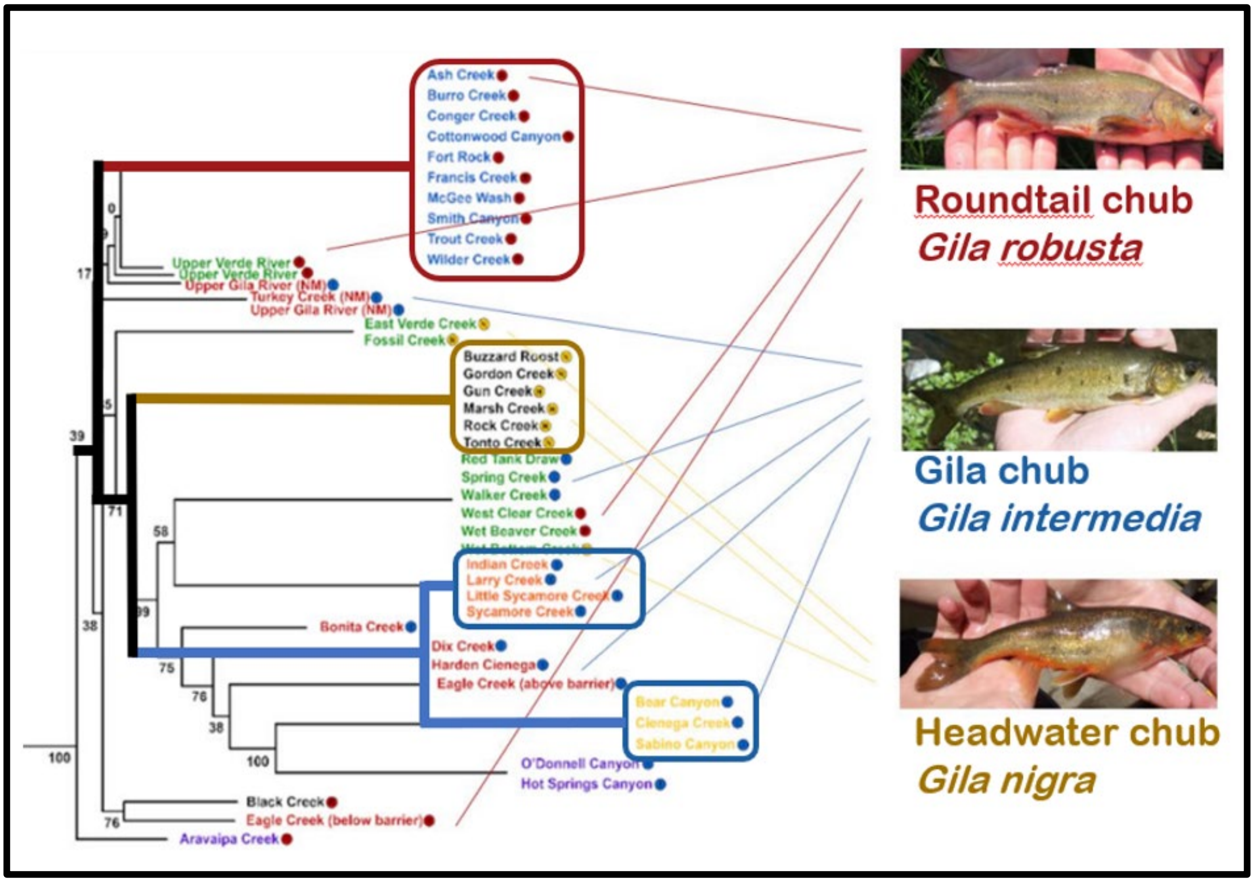

Given that we were required to redo the analysis in review, we also provide it here for interested readers. Given the genetic divergence of the LCR population is most likely to alter the outcome of the hierarchical AMOVA analysis comparing relative support for the nominal taxonomy compared to the geographic distinctions, we re-ran this analysis to determine if exclusion of LCR changes the outcome. We get essentially the same result from the AMOVA with or without LCR (modified Table 2, below). If anything, this subset of the data provides even less support for the nominal species (11.6 vs 22.8%) than when LCR is included (23.1 vs 30.9%). The same was true of the STRUCTURE analyses which were qualitatively similar and did not recover support for three nominal taxa with or without LCR. Thus, given that the divergence of LCR does not alter the outcome or interpretations of this study, we include all data throughout this manuscript as originally designed and do not exclude any samples from our analyses.

Source of Variation	Sum of Squares	Variance Components	Percentage Variation	Source of Variation	Sum of Squares	Variance Components	Percentage Variation
**Among species groups**	77920.64	93.83	11.64	**Among watershed groups**	191108.10	182.63	22.80
**Among streams within species groups**	284609.34	271.76	33.72	**Among streams within watershed groups**	171421.88	177.98	22.22
**Among individuals within streams**	194748.38	-41.55	-5.16	**Among individuals within streams**	256060.50	-41.55	-5.19
**Within individuals**	256060.50	481.85	59.79	**Within individuals**	243505.00	481.85	60.16

Modified Table 2 (LCR excluded): Analysis of molecular variance (AMOVA) testing alternate hypotheses using watersheds or nominal species within the *Gila robusta* complex as the unit of comparisons after removal of the most divergent population (LCR) from the dataset to test if watershed still explains more of the variation than nominal species.

Below we provide the code and detailed results of the DAPC for those who wish to repeat, modify or dig further into our analyses.

Gila DAPC

Cameron Walsh

For a tutorial on DAPC analyses (upon which this analysis is based), click here (https://adegenet.r-forge.r-project.org/files/tutorial-dapc.pdf).

Prepare environment

Prepare environment by setting working directory, setting random seed for reproducibility, loading required package(s) and data. Since the longest part of this step is the vcfR2genind conversion, I previously saved the genind output object in an Rdata file. I also had to correct some of the mislabelled samples. If running this for the first time, un-comment the single # lines or run them separately.

```
## Gila project
setwd("~/Desktop/Hawaii/research/Gila/")

### Set seed
set.seed(1284)

### Load packages and functions
# library(vcfR)
library(adegenet)
library(tidyverse)

### Convert data to required format
# vcf <- read.vcfR("chub.final.vcf")
# gila <- vcfR2genind(vcf)

### Fix mislabeled ids/samples
# ids_vector <- dimnames(gila@tab)[[1]]
# TRO2UPP <- grep("UPP_410|UPP_411|UPP_412|UPP_413|UPP_414", ids_vector)
# ids_vector[TRO2UPP] <- gsub("UPP", "TRO", ids_vector[TRO2UPP])
#
# ids_vector <- gsub("WAL_445", "UPP_445", ids_vector)

### Assign pops to genind
# gila@pop <- factor(gsub("[0-9].*|_[0-9].*", "", ids_vector))
# save(gila, file = "chub_genind.Rdata")
load("chub_genind.Rdata")
```

De novo cluster (k) selection

First, I run find.clusters() (k-means algorithm for DAPC) in the interactive setting to get a plot of BIC ~ number of clusters. This gives me a general idea of what to expect the “optimal K” might be. In this case, I use all principal components (PCs) as there is no reason not to keep all of them at this stage.

```
### Get initial graph of BIC ~ number of clusters
clusters4graph <- find.clusters(gila, pca.select = "nbEig", n.pca = nrow(gila@tab),
                                max.n.clust = 45)
```

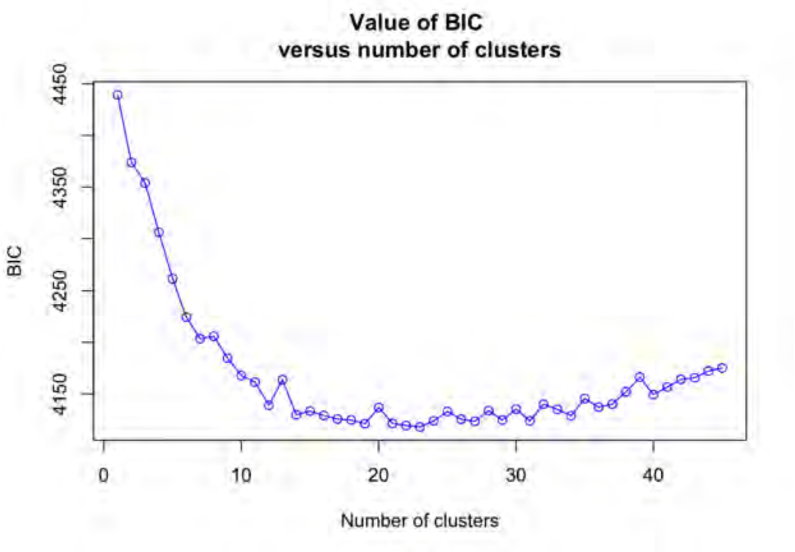


```
## Choose the number of clusters (>=2:
```

From the graph above, it looks like the minimum/lowest BIC score would be with 23 clusters (after which point there is a generally steady increase in BIC). This is likely to change slightly across different iterations. As it is along more or less of an asymptote, the “min” criterion for cluster selection may not be ideal (lower k would probably suffice). Similarly, there are several points where the BIC goes up (bumps) while it is still not near the minimum, so simple “goesup” selection criteria would not be ideal either. This leaves three other potential criteria to choose the “optimal” number of clusters: “diffNgroup”, “smoothNgoesup”, and “goodfit”. I run k selection using each of these criteria below.

```
### Get initial graph of BIC ~ number of clusters
diffNgroup_clusters <- find.clusters(gila, pca.select = "nbEig", n.pca = nrow(gila@tab),
                                     choose.n.clust = F, criterion = "diffNgroup",
                                     max.n.clust = 45)

smoothNgoesup_clusters <- find.clusters(gila, pca.select = "nbEig", n.pca = nrow(gila@tab),
                                        choose.n.clust = F, criterion = "smoothNgoesup",
                                        max.n.clust = 45)

goodfit_clusters <- find.clusters(gila, pca.select = "nbEig", n.pca = nrow(gila@tab),
                                  choose.n.clust = F, criterion = "goodfit",
                                  max.n.clust = 45)
```

It is worth noting that the “diffNgroup” method is recommended for users that are “unsure” about the various criteria. This method is also said to possibly be unstable when there are initial, very sharp decreases in the test statistic (BIC here). It may be informative that the high number of clusters chosen by this method essentially corresponds to nearly the number of streams sampled in this study (n=45). While this implies that the genetic variation among our samples can best be explained between these sampling groups, it does not provide much more information about them than we knew a priori. Both the “smoothNgoesup” and “goodfit” agree on k = 12, so I continue with that k as a compromise. I check the membership of each cluster with regards to the streams their samples come from later on while examining the relationships between clusters on the DAPC discriminant functions (DFs) .

```
sapply(list(diffNgroup_clusters, smoothNgoesup_clusters, goodfit_clusters),
       function(x)x$stat)
```

```
##     K=43     K=12     K=12
## 4158.805 4170.296 4158.650
```

First, I check whether the two sets of K=12 groups agree on their assignment of all (624) individuals in this dataset.

```
sort(smoothNgoesup_clusters$size)
```

```
##  [1]  15  30  30  30  36  45  49  60  61  72  86 110
```

```
sort(goodfit_clusters$size)
```

```
##  [1]  15  15  21  30  30  49  58  61  72  74  90 109
```

They do not. I move forward with the clusters made by “goodfit” since they had lower BIC.

DAPC PC selection and main mapping with *de novo* clusters

Now I run the DAPC analysis. This is said to benefit from using fewer principle components, so similar to the DAPC tutorial mentioned above and as recommended by the “Recommended standard reporting for DAPC analyses” in Box 2 of Miller et al. (2020) (http://www.nature.com/articles/s41437-020-0348-2), we use a-score and the optim.a.score() function on a preliminary DAPC to determine the optimum number of principle components to retain. 58 PCs were retained in the final DAPC, and all DFs were kept for further examination. The proportion of variance explained by the first four PCs is also reported.

```
prelim_dapc <- dapc(gila, goodfit_clusters$grp, var.contrib = T,
                    pca.select = "percVar", perc.pca = 90, pca.info = T,
                    n.da = 11)
a <- optim.a.score(prelim_dapc)$best
```

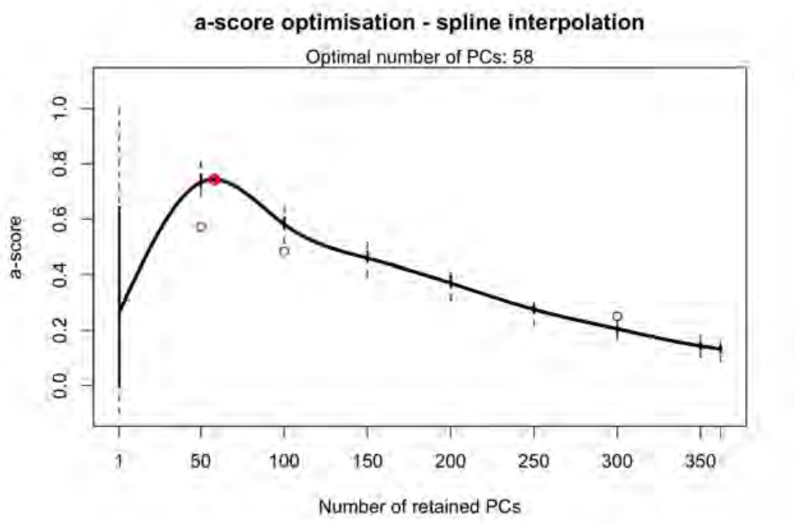


```
dapc <- dapc(gila, goodfit_clusters$grp, var.contrib = T,
             n.da = 11, n.pca = a, pca.info = T)
round(dapc$pca.eig[1:4]/sum(dapc$pca.eig)*100, digits = 2)
```

```
## [1] 10.98 10.33  5.50  5.37
```

The graphs below show the first 4 DFs. These are are analyzed and interpreted in detail below. Since one cluster is so far away on DF1, I also show a zoomed in version of the DF1–DF2 plot right underneath the original. Graphs showing all 11 DFs in succession (2D plots) can be found in the Appendix at the end of this document. The number of PCs retained (PC scree plot) is only shown on the first plot.

```
scatter(dapc, clab = 0.5,
        legend = T, posi.leg = "topleft",
        scree.da = T, posi.da = "topright",
        scree.pca = T, posi.pca = "bottomright")
```

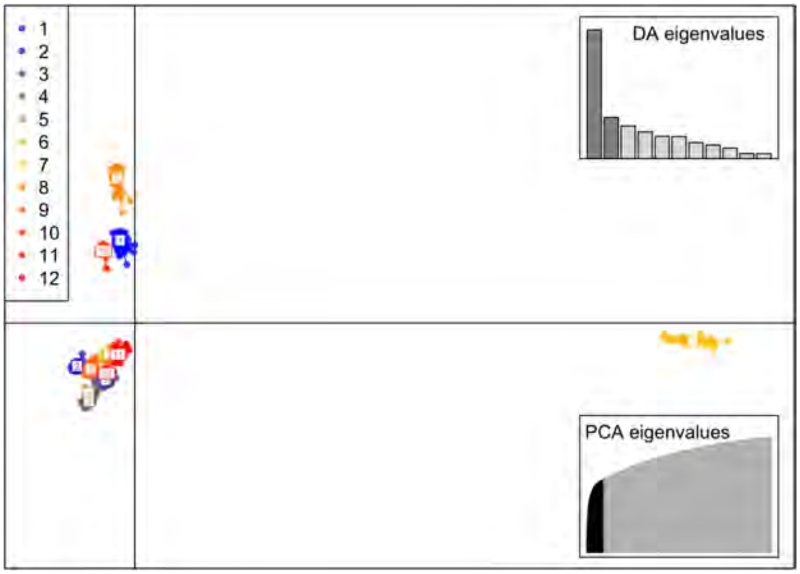


```
scatter(dapc, clab = 0.5,
        legend = T, posi.leg = "topleft",
        scree.da = T, posi.da = "topright",
        xlim = c(-15, 15))
```

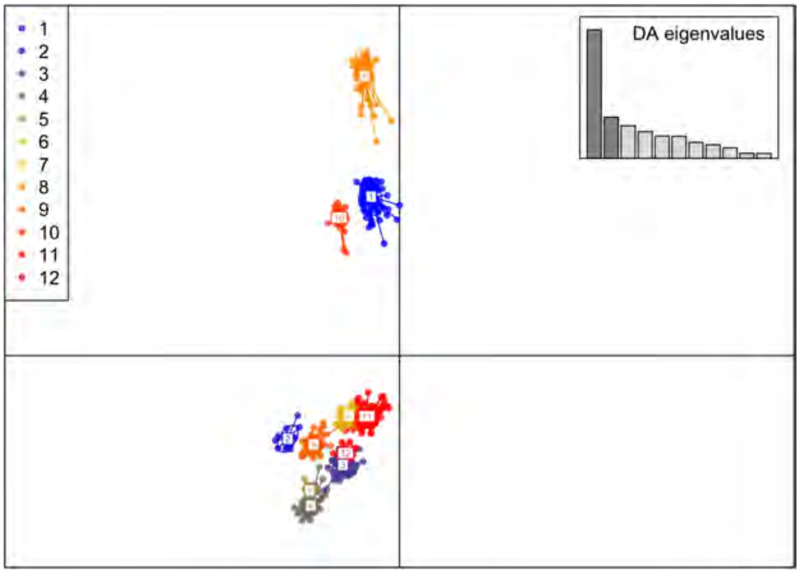


```
scatter(dapc, xax = 3, yax = 4, clab = 0.5,
        legend = T, posi.leg = "bottomleft",
        scree.da = T, posi.da = "topright")
```

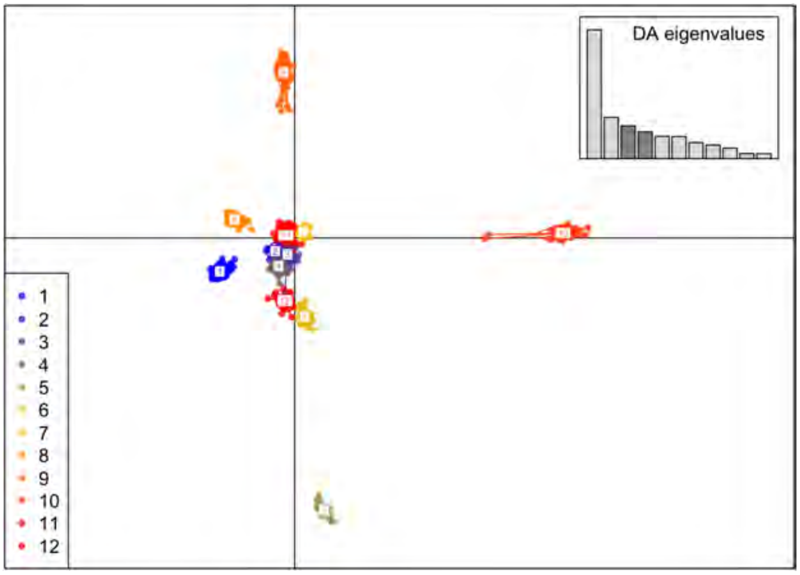

Here is a nice way to save some plots of DFs 1-4 with the help of magick.

```
png("A.png", width = 6.5, height = 4, units = "in", res = 300)
scatter(dapc, legend = F,
        clab = 0.625, cellipse = 0, cstar = 0,
        scree.da = T, posi.da = "topright",
        scree.pca = T, posi.pca = "bottomright")
dev.off()
```

```
## quartz_off_screen
##                 2
```

```
png("B.png", width = 6.5, height = 4, units = "in", res = 300)
scatter(dapc, xax = 3, yax = 4,
        clab = 0.625, cellipse = 0, cstar = 0,
        legend = T, posi.leg = "bottomleft",
        scree.da = T, posi.da = "topright")
dev.off()
```

```
## quartz_off_screen
##                 2
```

```
library(magick)
figureA <- image_read("A.png")
figureB <- image_read("B.png")

joint_figure <- image_annotate(image_append(c(figureA, figureB), stack = T),
                               "A\nB", size = 150, gravity = "west", location = "+25+")
image_write(joint_figure, path = "DAPC_DFs.png", format = "png")
```

Closer look at DF1

The first DF strongly separates group 7 from all other clusters. Even zoomed in towards the middle we see that all other clusters are still close together and overlapping.

```
scatter(dapc, 1, 1)
```

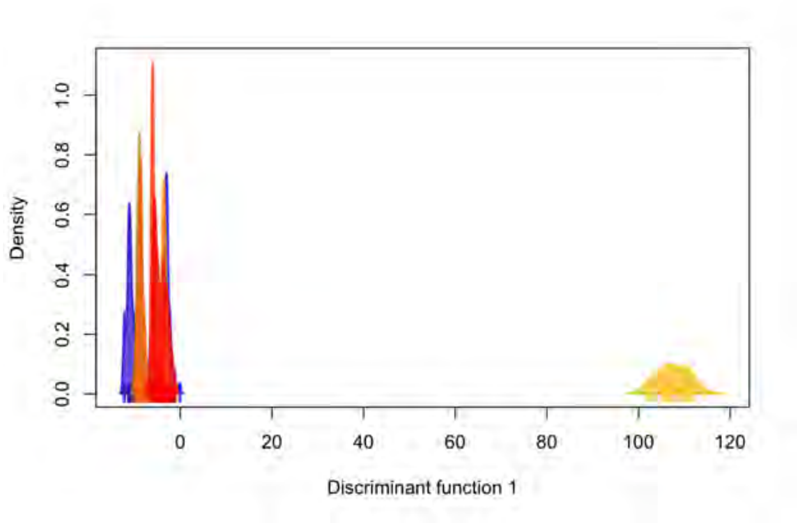

Cluster 7 contains all samples from the LCR watershed (considered *G. robusta*[?]), including 15 from ECLR (East Clear Creek) and 15 from CHC (Chevelon Creek).

```
group_7_members <- droplevels(sort(gila@pop[which(goodfit_clusters$grp==7)]))
summary(group_7_members)
```

```
##  CHC ECLR
##   15   15
```

I check whether this large difference on DF1 between cluster 7 and the others is caused by particular loci. The large eigenvalue for DF1 appears to be caused by many loci.

```
contrib <- loadingplot(dapc$var.contr, axis = 1, thres = 4.7e-4, lab.jitter = 10)
```

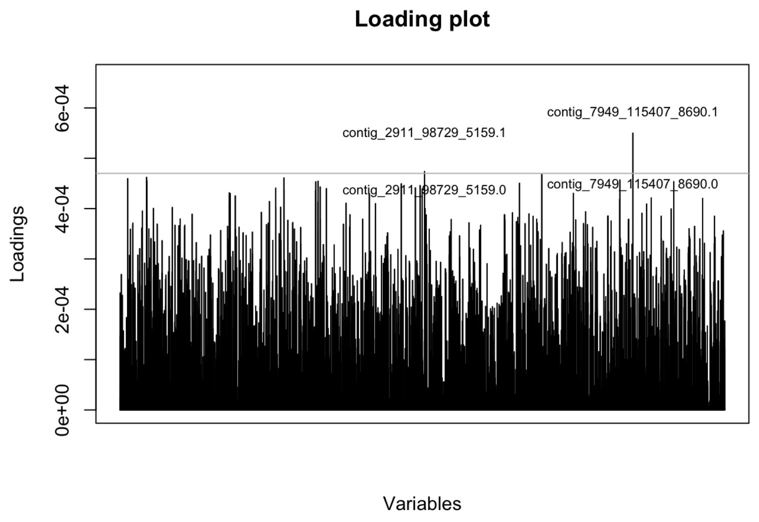

While the locus with the largest loading is variable in many clusters (while fixed in some), the locus with the second largest loading is fixed in cluster 7 for an allele opposite to one that is nearly fixed across all other clusters.

```
loc_list <- seploc(gila)

allele_freqs <- function(ind_mat) {
  apply(ind_mat, 2, function(e) tapply(e, goodfit_clusters$grp, mean, na.rm = TRUE))/2
}

freq_list <- lapply(loc_list, function(x) allele_freqs(tab(x)))

plot_freqs <- function(allele_freq_mat, ref_alt = c("?", "?")) {
  allele_freq_mat <- allele_freq_mat[order(allele_freq_mat[,1], decreasing = T),
                                     order(colnames(allele_freq_mat))]
  colnames(allele_freq_mat) <- ref_alt # check vcf
  {matplot(allele_freq_mat, type = "b", xaxt = "n",
          pch = colnames(allele_freq_mat), cex = 1,
          xlab = "cluster", ylab = "allele frequency")
    axis(side = 1, at = 1:nrow(allele_freq_mat), labels = rownames(allele_freq_mat))}
}

plot_freqs(freq_list$contig_7949_115407_8690, c("T", "A")) # check vcf for ref_alt
```

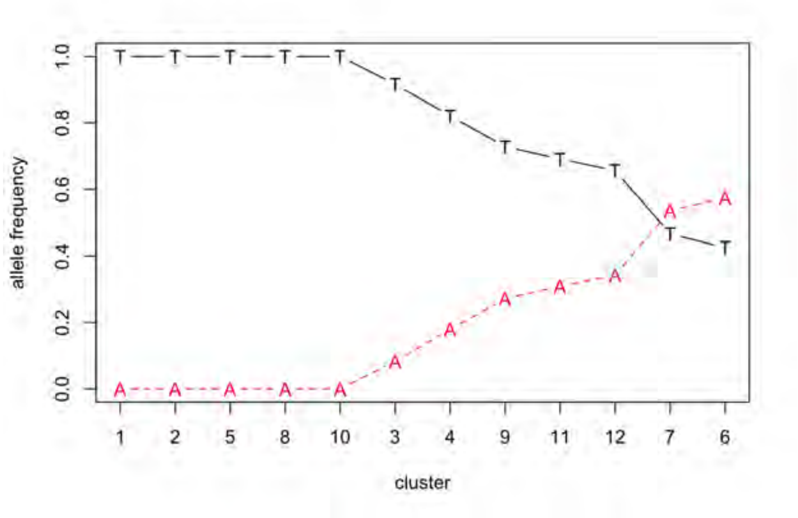


```
plot_freqs(freq_list$contig_2911_98729_5159, c("C", "T"))
```

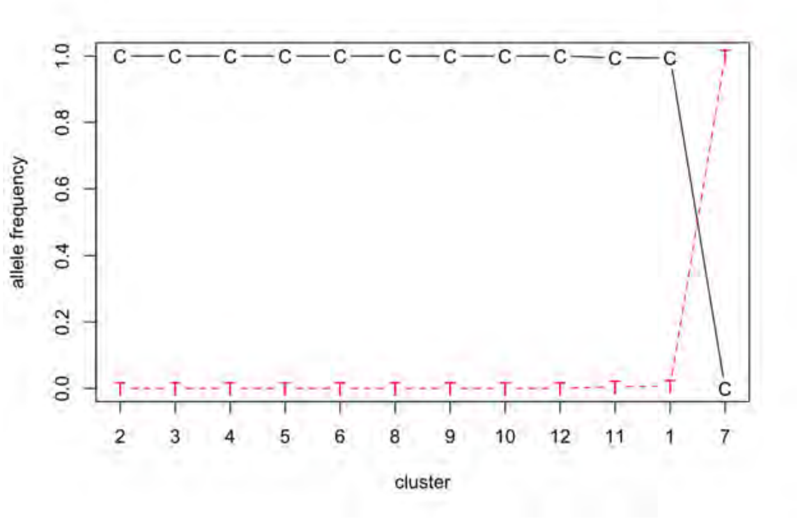


```
freq_list$contig_2911_98729_5159
```

```
##    contig_2911_98729_5159.0 contig_2911_98729_5159.1
## 1                 0.9940476              0.005952381
## 2                 1.0000000              0.000000000
## 3                 1.0000000              0.000000000
## 4                 1.0000000              0.000000000
## 5                 1.0000000              0.000000000
## 6                 1.0000000              0.000000000
## 7                 0.0000000              1.000000000
## 8                 1.0000000              0.000000000
## 9                 1.0000000              0.000000000
## 10                1.0000000              0.000000000
## 11                0.9947368              0.005263158
## 12                1.0000000              0.000000000
```

Fixed private alleles in clusters

This begs the question as to whether there are fixed differences unique to certain clusters (i.e. fixed private alleles). There are 297 fixed differences unique to cluster 7. The only other cluster with fixed private alleles is cluster 10 which has six. None of these fixed private alleles in cluster 10 were on the same contig.

```
cluster_fixed_differences <- list()
for(a in seq_along(goodfit_clusters$size)) {
cluster_fixed_differences[a] <- list(names(which(!is.na(sapply(freq_list,
                                                                 function(x) ifelse(x[a,1]==1 && sum(x[,1])==1 |
                                  x[a,2]==1 && sum(x[,2])==1, x, NA))))))
}
total_fixed_diffs <- sapply(cluster_fixed_differences, length)
names(total_fixed_diffs) <- paste0("C", seq_along(total_fixed_diffs))
total_fixed_diffs
```

```
##  C1  C2  C3  C4  C5  C6  C7  C8  C9 C10 C11 C12
##   0   0   0   0   0   0 297   0   0   6   0   0
```

```
cluster_fixed_differences[[10]]
```

```
## [1] "contig_11171_16987_474"  "contig_3222_200284_5552"
## [3] "contig_3341_436570_5711" "contig_450_186902_6874"
## [5] "contig_8210_29418_8792"  "contig_980_59361_9577"
```

There are some contigs and/or scaffolds with multiple fixed differences unique to cluster 7. The graph below shows the number of contigs that have n cluster 7 fixed private alleles. Although most contigs with a fixed allele unique to cluster 7 contain no more than one such locus, up to five can be found on a single contig.

```
group_7_fixed <- data.frame(uniqueID = cluster_fixed_differences[[7]])
group_7_fixed$contig_scaff <- gsub("_[0-9]*_[0-9]*_[0-9]*", "", group_7_fixed$uniqueID)
group_7_fixed$section <- as.numeric(gsub("contig_|scaffold_|_[0-9]*_[0-9]*$", "", group_7_fixed$uniqueID))
group_7_fixed <- group_7_fixed[with(group_7_fixed, order(contig_scaff, section)),]
group_7_fixed$cs_section <- paste0(group_7_fixed$contig_scaff, "_", group_7_fixed$section)
group_7_fixed$cs_section <- factor(group_7_fixed$cs_section, levels = unique(group_7_fixed$cs_section))

fixed_by_section <- group_by(group_7_fixed, cs_section) %>%
  summarize(.groups = "keep",
            n = length(cs_section))

ggplot(data = fixed_by_section) +
  geom_bar(aes(x = n)) +
  theme_classic()
```

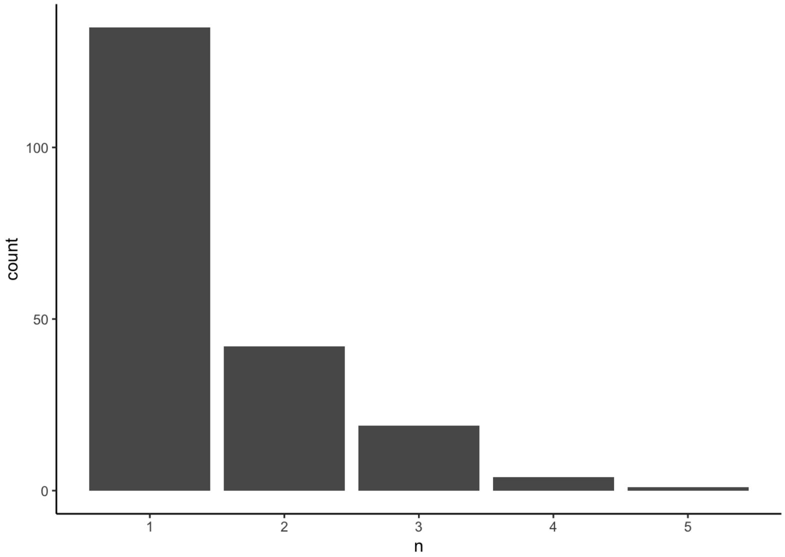

The contigs and/or scaffolds with at least four fixed differences unique to cluster 7 are labelled in this plot of all contigs (with at least one fixed difference unique to cluster 7) ordered by their “position” in the genome (contig numbers then scaffold numbers).

```
multivars_cs_section <- pull(fixed_by_section[which(fixed_by_section$n>3), "cs_section"])

ggplot(group_7_fixed, aes(x = cs_section)) +
  geom_bar(width = 1) +
  geom_text(stat = "count",
            aes(label = ifelse(cs_section %in% multivars_cs_section,
                               as.character(cs_section), "")), vjust = -1, size = 3) +
  scale_y_continuous(limits = c(0, 5.5), expand = c(0,0)) +
  theme_classic() +
  theme(axis.text.x = element_blank(),
        axis.ticks.x = element_blank(),
        axis.title.x = element_blank())
```

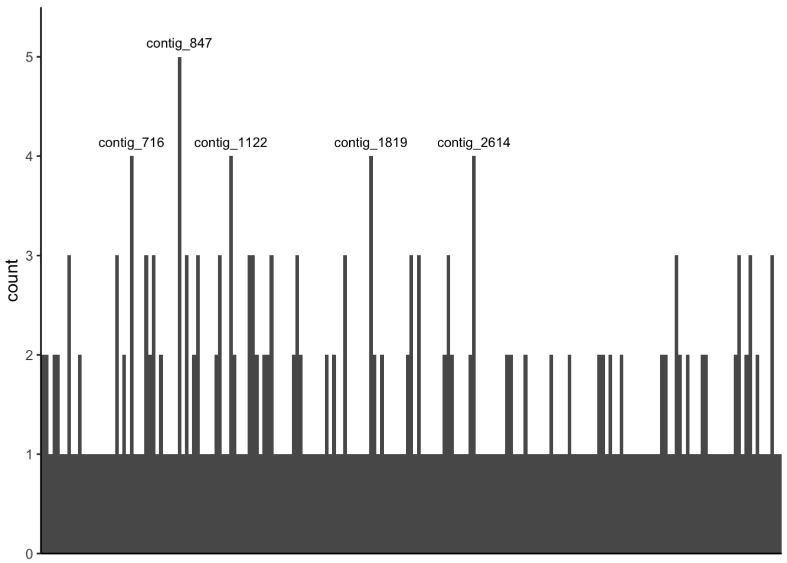

It looks like some of these fixed SNPs are quite close together on their contigs (e.g. the four sites on contig 1122), but many are spread out too. I checked these contigs and loci in the annotation, only one coding sequence overlapped and it was in an uncharacterized zebrafish gene).

```
lapply(multivars_cs_section, function(x) as.character(group_7_fixed[which(group_7_fixed$cs_section %in% x), "uniqueID"]))
```

```
## [[1]]
## [1] "contig_716_429538_8262" "contig_716_529019_8263" "contig_716_667184_8267"
## [4] "contig_716_667252_8268"
##
## [[2]]
## [1] "contig_847_507973_8973"  "contig_847_508009_8974"
## [3] "contig_847_508033_8975"  "contig_847_724295_8980"
## [5] "contig_847_1258360_8984"
##
## [[3]]
## [1] "contig_1122_686374_514" "contig_1122_686381_515" "contig_1122_686384_516"
## [4] "contig_1122_686395_517"
##
## [[4]]
## [1] "contig_1819_103437_2775" "contig_1819_166321_2777"
## [3] "contig_1819_166354_2778" "contig_1819_204497_2782"
##
## [[5]]
## [1] "contig_2614_38580_4499"   "contig_2614_1076785_4522"
## [3] "contig_2614_1520404_4524" "contig_2614_2028971_4533"
```

Closer look at DFs 2–4

Back to the DAPC. The second DF has clusters 1, 8, & 10 situated away from where the others clusters lump together.

```
scatter(dapc, 2,2)
```

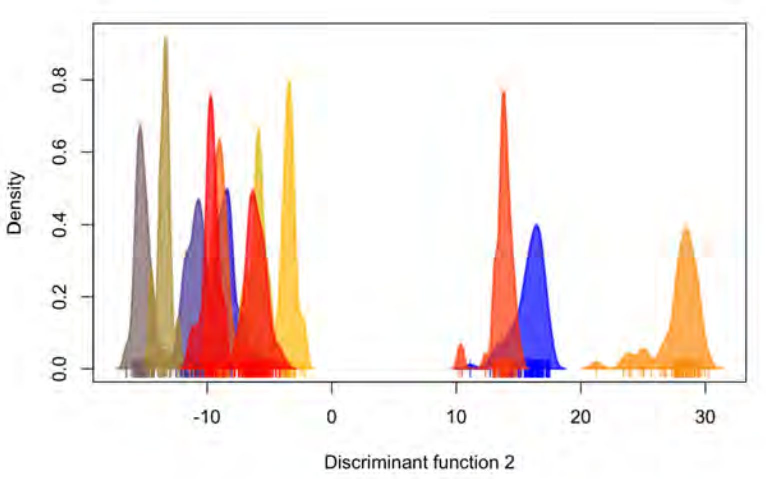

Cluster 8 is furthest from most clusters. It contains 61 individuals: all samples from ASH, FOR, MCG, and TRO—all of which are in the Bill Williams River watershed and consequently considered *G. robusta*.

```
group_8_members <- droplevels(sort(gila@pop[which(goodfit_clusters$grp==8)]))
summary(group_8_members)
```

```
## ASH FOR MCG TRO
##  13  15  15  18
```

Cluster 1 contains 90 individuals: all remaining samples from the Bill Williams River watershed. This includes the samples from BUR, CON, COTCAN, FRAN, SMITHC & WLDR (again all *G. robusta*)

```
group_1_members <- droplevels(sort(gila@pop[which(goodfit_clusters$grp==1)]))
summary(group_1_members)
```

```
##    BUR    CON COTCAN   FRAN SMITHC   WLDR
##     15     15     15     15     15     15
```

Cluster 10 contains all 49 samples from the Agua Fria River watershed (IND, LAR, LSYC, SYC), considered to be *G. intermedia*.

```
group_10_members <- droplevels(sort(gila@pop[which(goodfit_clusters$grp==10)]))
summary(group_10_members)
```

```
##  IND  LAR LSYC  SYC
##   15   14    5   15
```

DF2 therefore distinguishes all samples from the two most western watersheds (Bill Williiams River and Agua Fria River) from all other samples. This DF therefore groups clusters containing different species as more similar to each other than they are to all other samples, including other samples of their own purported species.   Cluster 10 had some fixed private alleles, so the loci loadings may be interesting to look at for this DF as well. Their appears to be greater variance in the loading of loci in this DF, such that some loci have much larger loadings than others or than average. I take a closer look at the loci with the two largest loadings in the Appendix. These two loci were similar to the two examined with DF1, and neither were cluster 10 fixed private alleles.

```
contrib <- loadingplot(dapc$var.contr, axis = 2, thres = 0.00125, lab.jitter = 10)
```

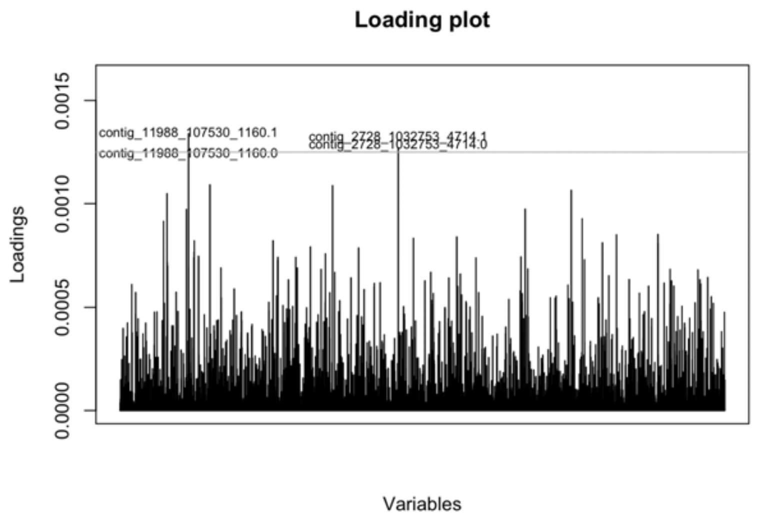

DF3 again largely distinguishes clusters 1, 8, and 10 from the others. However, this time cluster 10 is distinguished from all others clusters to one side, while clusters 1 and 8 are separated from all remaining clusters to the other side. DF3 therefore largely differentiates the Agua Fria River watershed from the Bill Williams watershed (the two that were grouped together in DF2).

```
scatter(dapc, 3,3)
```

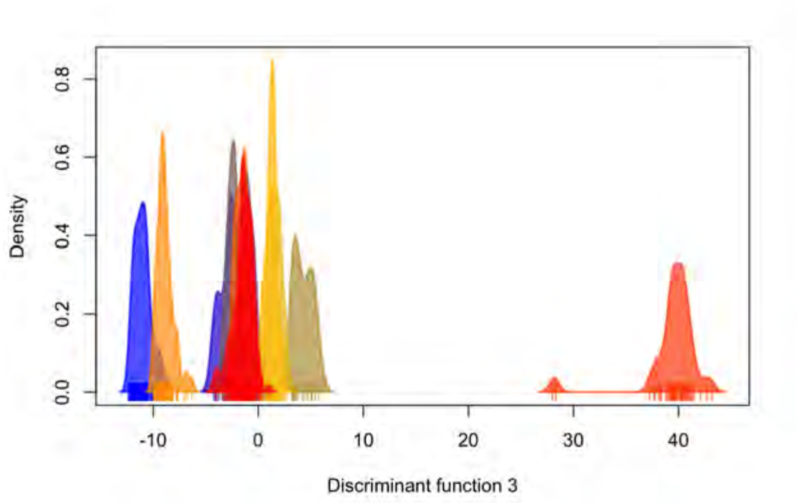

The locus loadings of DF3 appear to be intermediate relative to the previous two DFs. None are as large as the largest two examined with DF2, but there is still quite a large difference between some of the smallest and largest. The two largest are also not any of the fixed private alleles of cluster 10, and are examined further in the Appendix.

```
contrib <- loadingplot(dapc$var.contr, axis = 3, thres = 0.00095, lab.jitter = 10)
```

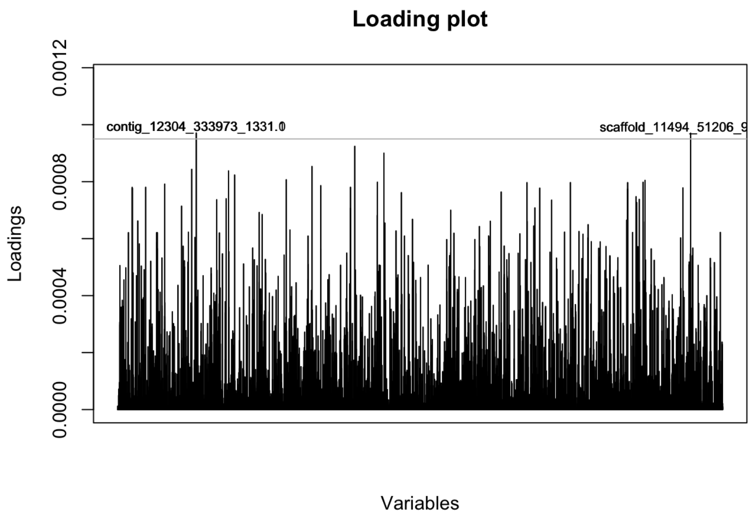

DF4 separates cluster 5 and cluster 9 on opposite sides of each other, with all other clusters grouped between them. On the same side of the DF as cluster 5 are clusters 6 and 12. Their distribution on DF4 has little to no overlap with the main group of clusters, even if they are not particularly far away from the main group relative to cluster 5.

```
scatter(dapc, 4, 4)
```

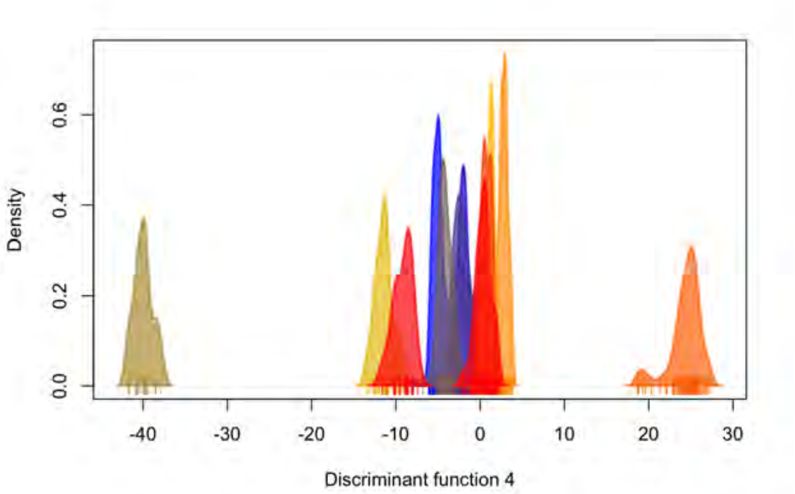

Cluster 5 contains all samples from WCC in the Verde River watershed (considered *G. robusta*)

```
group_5_members <- droplevels(sort(gila@pop[which(goodfit_clusters$grp==5)]))
summary(group_5_members)
```

```
## WCC
##  15
```

Cluster 9 contains 72 samples from the Salt River watershed considered to be *G. nigra*: all samples from BUZ, GOC, GUN, MAR, ROC, and TONTO. The only Salt River samples not in this group are those from BLACK.

```
group_9_members <- droplevels(sort(gila@pop[which(goodfit_clusters$grp==9)]))
summary(group_9_members)
```

```
##   BUZ   GOC   GUN   MAR   ROC TONTO
##    15     3    15    14    15    10
```

Cluster 6 contains all 30 samples from two locations in the Verde River watershed: RTD and SPR (said to be *G. intermedia*).

```
group_6_members <- droplevels(sort(gila@pop[which(goodfit_clusters$grp==6)]))
summary(group_6_members)
```

```
## RTD SPR
##  15  15
```

Cluster 12 (like cluster 6) contains all 21 samples from two locations in the Verde River watershed. Those from WAL are said to be *G. intermedia*, while those from WTBV are said to be *G. robusta*.

```
group_12_members <- droplevels(sort(gila@pop[which(goodfit_clusters$grp==12)]))
summary(group_12_members)
```

```
##  WAL WTBV
##    6   15
```

DF4 thus largely separates the adjacent**[?]** Verde River and Salt Lake sampling locations from each other. The three Verde River clusters mentioned on one side of this DF4 together contain both *G. robusta* and *G. intermedia* (cluster 12 contains both on its own).

The eigenvalues start to flatten out more after DF4. Some comments about the other DFs, as well as which clusters they discriminate, are in the first part of the Appendix which covers the membership of clusters not yet discussed.

Main results summary

To recap overall results, 12 clusters were selected by k-means for DAPC analysis. These clusters contained 15—109 individuals from 1—8 streams in 1—4 watersheds. Three clusters contained individuals that would be considered different species based on their sampling locations, with one of those three clusters containing all three putative species.

HSC was the only stream to not group entirely into one cluster: a single sample from there was assigned to cluster 3 rather than with the other 14 samples from its stream in cluster 4. This means that only 1 out of 624 total samples in this study did not cluster with the rest of its stream.

Table 1. Summary of cluster membership by stream, watershed, and purported species

cluster	nSamples	nStreams	stream names	nWatersheds	watershed names	nSpecies	species names
1	90	6	BUR, CON, COTCAN, FRAN, SMITHC, WLDR	1	Bill Williams River	1	*G. robusta*
2	15	1	CIEN	1	Santa Cruz River	1	*G. intermedia*
3	74	6	BON, DIX, EAG, HAR, **HSC**, WTBM	3	Gila River, San Pedro River, Verde River	2	*G. intermedia*, *G. nigra*
4	58	4	**HSC**, ODON, BEAR, SAB	2	San Pedro River, Santa Cruz River	1	*G. intermedia*
5	15	1	WCC	1	Verde River	1	*G. robusta*
6	30	2	RTD, SPR	1	Verde River	1	*G. intermedia*
7	30	2	ECLR, CHC	1	LCR	1	*G. robusta[?]*
8	61	4	ASH, FOR, MCG, TRO	1	Bill Williams River	1	*G. robusta*
9	72	6	BUZ, GOC, GUN, MAR, ROC, TONTO	1	Salt River	1	*G. nigra*
10	49	4	IND, LAR, LSYC, SYC	1	Agua Fria River	1	*G. intermedia*
11	109	8	ARA, BLACK, EAGLE, EVERDE, FC, GILA, TURK, UPP	4	San Pedro River, Salt River, Gila River, Verde River	3	*G. intermedia*, *G. nigra*, *G. robusta*
12	21	2	WAL, WTBV	1	Verde River	1	*G. intermedia*, *G. robusta*

Table 2. Number of clusters containing samples from each watershed and species

watershed or species	number of clusters samples are represented in
Agua Fria River	1
Bill Williams River	2
Gila River	2
LCR	1
Salt River	2
San Pedro River	3
Santa Cruz River	2

watershed or species	number of clusters samples are represented in
Verde River	5
G. intermedia	7
G. nigra	3
G. robusta	6* (including highly-differentiated LCR)

Interpretation

The first part of characterizing genetic variance in this analysis involved using k-means to create de novo clusters. At this stage, samples of multiple putative species were grouped into the same cluster multiple times (as seen in the Tables above that summarize the descriptions of cluster membership in the main analysis and the Appendix).

The DAPC analysis itself mapped clusters onto different axes based on their similarity. The LCR (*G. robusta*[?]) samples are by far the most distinct of the 624 in this study.

A group of three clusters containing all samples from the Bill Williams River watershed and the Agua Fria River watershed were then grouped together away from all other samples on the second DF (DF2).

This DF2 result contradicts with the three species hypothesis in multiple ways: 1. The three clusters separated from all others on DF2 contains two different putative species: Bill Williams River *Gila* are said to be *G. robusta*, while the Agua Fria River *Gila* are said to be *G. intermedia*. 2. The clusters in the main DF2 group contain representatives from all three putative species. 3. A simpler explanation of DF2 is that these three separated clusters contain all samples from the two westernmost watersheds sampled in this study.

The third DF then primarily differentiates between these two watershed groups.

The fourth DF produces yet another geographic split in which multiple putative species are lumped together. The majority of samples from the Salt River watershed (cluster 9, putatively *G. nigra*) are split apart from the majority of samples from the Verde River watershed (cluster 5, putatively *G. robusta*; cluster 6, putatively *G. intermedia*; and cluster 12, ~2/3 *G. robusta* and ~1/3 *G. intermedia*).

Overall, both the de novo clustering and DAPC analyses consistently group samples of different putative species together, while segregating watersheds and streams much less frequently.

References

Jombart, T., & Collins, C. (2015). A tutorial for Discriminant Analysis of Principal Components (DAPC) using adegenet 2.0.0. Imperial College, London, United Kingdom.

Miller, J. M., Cullingham, C. I., & Peery, R. M. (2020). The influence of a priori grouping on inference of genetic clusters: simulation study and literature review of the DAPC method. Heredity, 125(5), 269-280.

Appendix

Membership of remaining clusters

Cluster 2 contains all 15 samples from CIEN in the Santa Cruz River watershed (*G. intermedia*). Cluster 2 groups and overlaps with most other clusters in the middle of all other DFs except DF7 which distinguishes it from all others.

```
group_2_members <- droplevels(sort(gila@pop[which(goodfit_clusters$grp==2)]))
summary(group_2_members)
```

```
## CIEN
##   15
```

```
scatter(dapc, 7, 7)
```

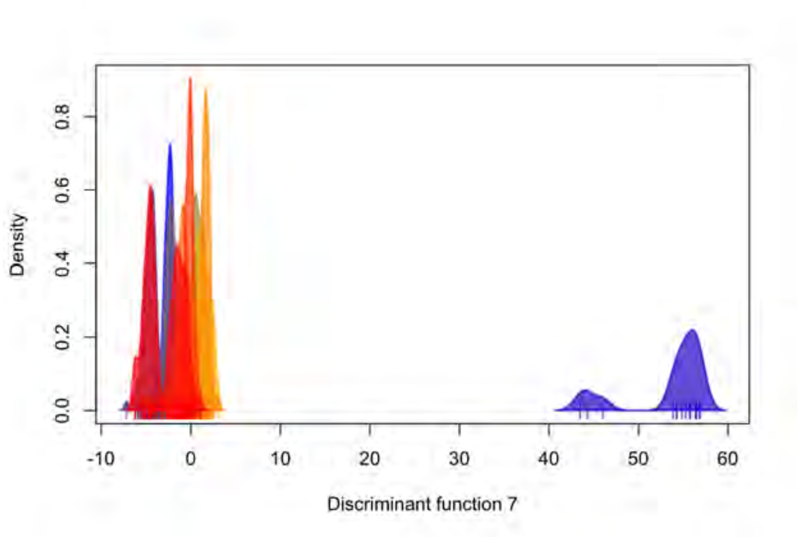

Cluster 3 contains 74 individuals. All samples from BON, DIX, EAG, and HAR—which are 4 of the 7 sampled locations in the Gila River watershed—are in this cluster. These samples, along with one individual from HSC in the San Pedro River watershed also in this cluster, are considered *G. intermedia*. Finally, all samples from WTBM in the Verde River watershed (considered *G. nigra*) are also in this cluster. This cluster broadly overlaps with most others in all DFs other than DF10, where it groups away from all others on its own (albeit the eigenvalue for this DF is very

small). However, individuals from cluster 3 map quite far outside its own intertia ellipse and (and sometimes in the ellipses of other non-overlapping clusters) on DFs 4–5 and 8–10. For example, there are samples from cluster 3 that reach out into clusters 4 and 12 on DFs 4–5. Cluster 4 contains 14/15 of the HSC samples, so it is likely that the single HSC sample in cluster is the one grouping with them in these DFs.

```
group_3_members <- droplevels(sort(gila@pop[which(goodfit_clusters$grp==3)]))
summary(group_3_members)
```

```
##  BON  DIX  EAG  HAR  HSC WTBM
##   15   14   15   14    1   15
```

```
scatter(dapc, 10, 10)
```

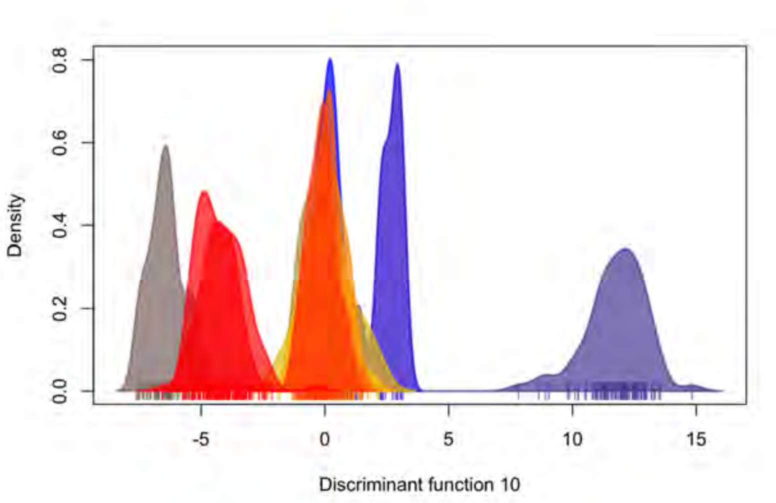

Cluster 4 contains 58 individuals. From the San Pedro River watershed are the remaining 14 HSC samples and all samples from ODON. The only other samples from the San Pedro River watershed in this study are those from ARA and the single HSC sample assigned to cluster 3. All samples from BEAR and SAB are also in this cluster, which along with the CIEN samples from cluster 2, make up all samples from the Santa Cruz River watershed in this study. All considered samples in this cluster would be considered *G. intermedia*. While slightly overlapping with other clusters in the middle of DF2, cluster 4 was mapped farthest (away from clusters 1, 8, and 10) to the left. Cluster 4 groups off on its own (with a single sample from cluster 3 overlapping as mentioned above) on DF5. Similar to DF2, clusters 4 and 5 group furthest to one side on DF8, except this time there is no overlap between them and other clusters.

```
group_4_members <- droplevels(sort(gila@pop[which(goodfit_clusters$grp==4)]))
summary(group_4_members)
```

```
## BEAR  HSC ODON  SAB
##   15   14   14   15
```

```
scatter(dapc, 5, 5)
```

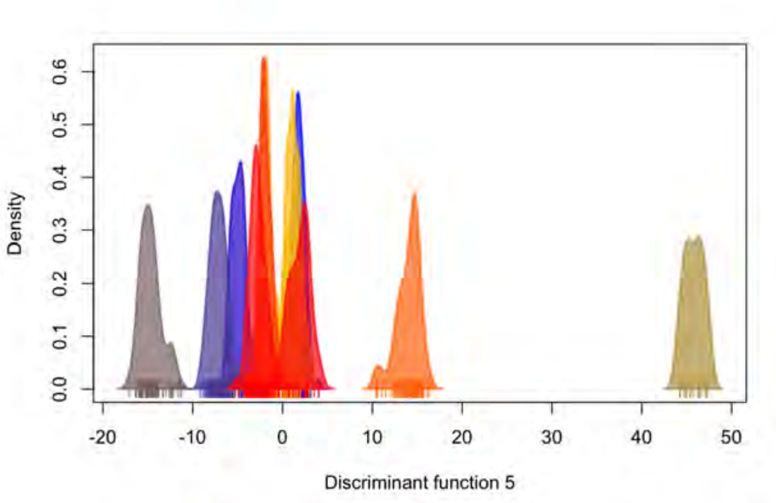

Cluster 11 is the largest in this study with 109 samples. It contains samples from the San Pedro River watershed (ARA: *G. robusta*), the Salt River watershed (BLACK: *G. robusta*), the Gila River watershed (EAGLE: *G. robusta*; GILA, TURK: *G. intermedia*), and the Verde River watershed (EVERDE, FC: *G. nigra*; UPP: *G. robusta*). This cluster contains samples from four watersheds, purported to be three different species. With so many samples, streams, watersheds and putative species in this cluster, it nearly always overlaps with other clusters in the middle of all DFs. The exception to this is in DF9 (which is the most spread out) where it maps farthest to the right and does not overlap with other clusters.

```
group_11_members <- droplevels(sort(gila@pop[which(goodfit_clusters$grp==11)]))
summary(group_11_members)
```

```
##    ARA  BLACK  EAGLE EVERDE     FC   GILA   TURK    UPP 
##     15     14     15     15     15     15      9     11
```

```
scatter(dapc, 9, 9)
```

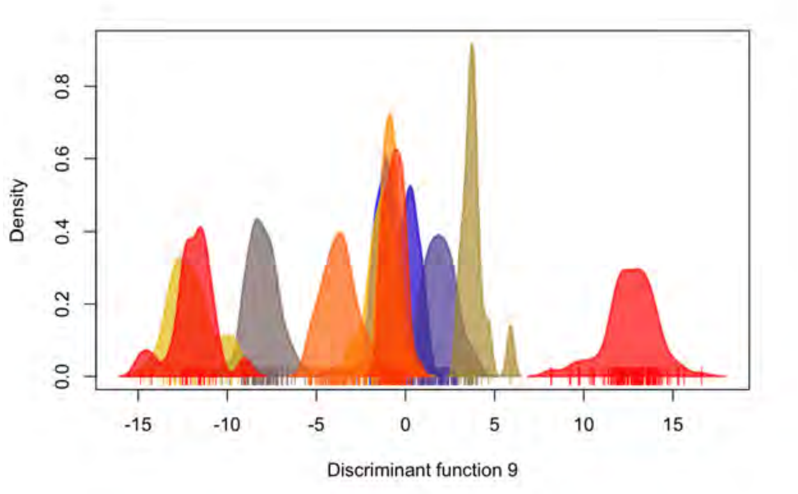

DF2 and DF3 large loading loci

The DF2 loci with the largest loading look similar to the two loci examined with the first DF. The locus with the largest loading is widely variable across many populations. It is fixed for one allele in clusters 2 & 10, at >99% frequency in cluster 1, and >90% frequency in cluster 8. The locus with the second largest loading also had one allele fixed in clusters 7, 8 & 10, but only ~35% frequency in cluster 1. This locus was only variable in one other cluster (11; ~3.5%), and fixed for the opposite allele to clusters 7, 8 & 10 in all other clusters. No loci had alleles that were unique to and fixed in clusters 1, 8, 10 together.

```
plot_freqs(freq_list$contig_11988_107530_1160, c("C", "T"))
```

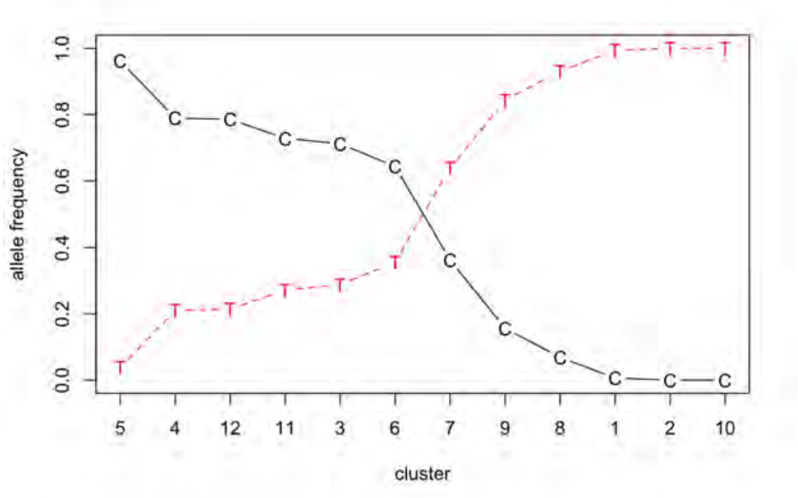


```
plot_freqs(freq_list$contig_2728_1032753_4714, c("G", "A"))
```

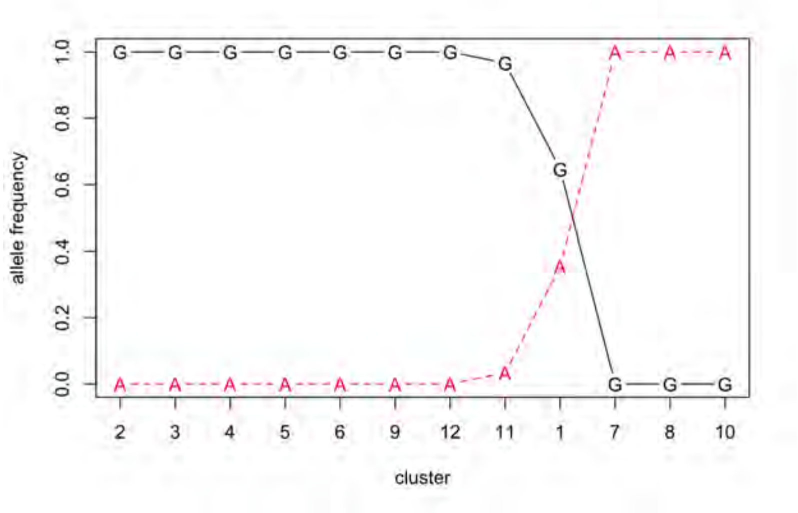


```
list(names(which(!is.na(sapply(freq_list,
                                function(x) ifelse(sum(x[c(1, 8, 10),1])==3 &&
                                                     sum(x[,1])==3 |
                                                     sum(x[c(1, 8, 10),2])==3 &&
                                                     sum(x[,2])==3, x, NA))))))
```

```
## [[1]]
## character(0)
```

The loci with the largest DF3 loadings were not among the ones discovered earlier to be fixed and private in cluster 10. Instead, one loci was fixed for one allele in cluster 10 but fixed for the other allele in clusters 2, 8 & 9 (and at >99% in cluster 1). The other is a locus with an allele that is fixed in half of the clusters, and at no lower than 90% frequency in any cluster but 10 for which it was at only ~2.5% frequency.

```
plot_freqs(freq_list$contig_12304_333973_1331, c("C", "A"))
```

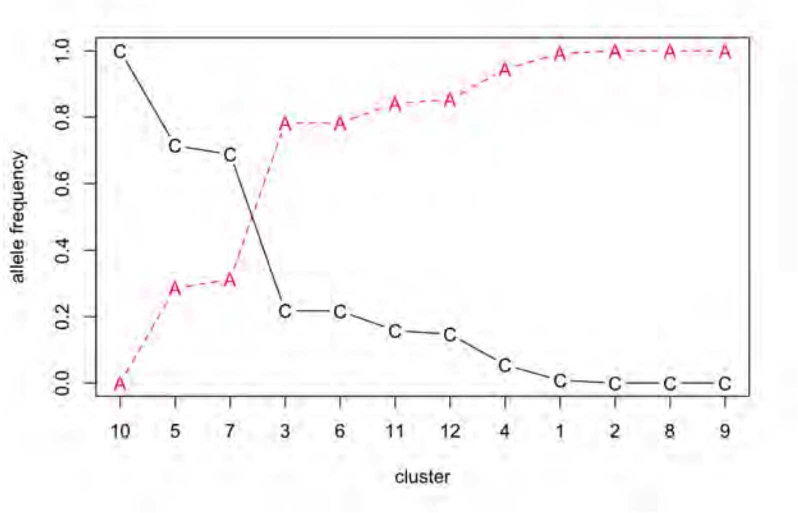


```
plot_freqs(freq_list$scaffold_11494_51206_9703, c("C", "T"))
```

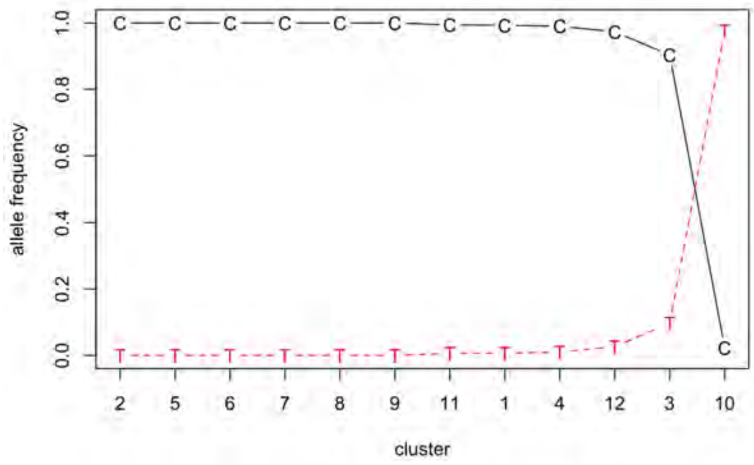

All 2D DF plots

```
scatter(dapc, clab = 0.5,
        legend = T, posi.leg = "topleft",
        scree.da = T, posi.da = "topright",
        scree.pca = T, posi.pca = "bottomright")
```

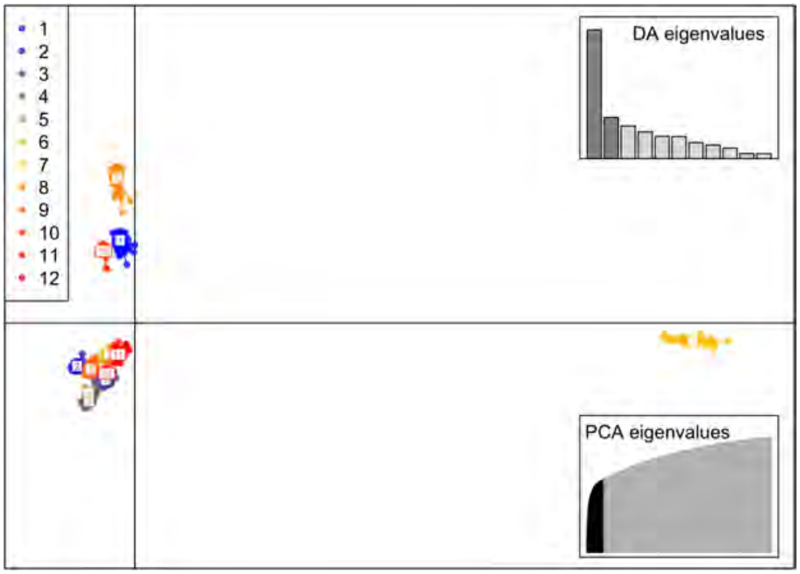


```
scatter(dapc, clab = 0.5,
        legend = T, posi.leg = "topleft",
        scree.da = T, posi.da = "topright",
        xlim = c(-15, 15))
```

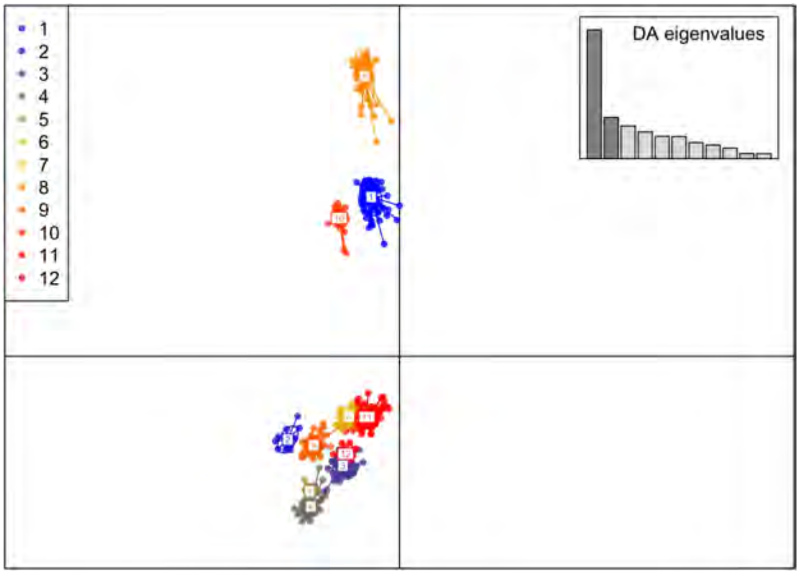

```
scatter(dapc, xax = 2, yax = 3, clab = 0.5,
        legend = T, posi.leg = "topleft",
        scree.da = T, posi.da = "topright")
```

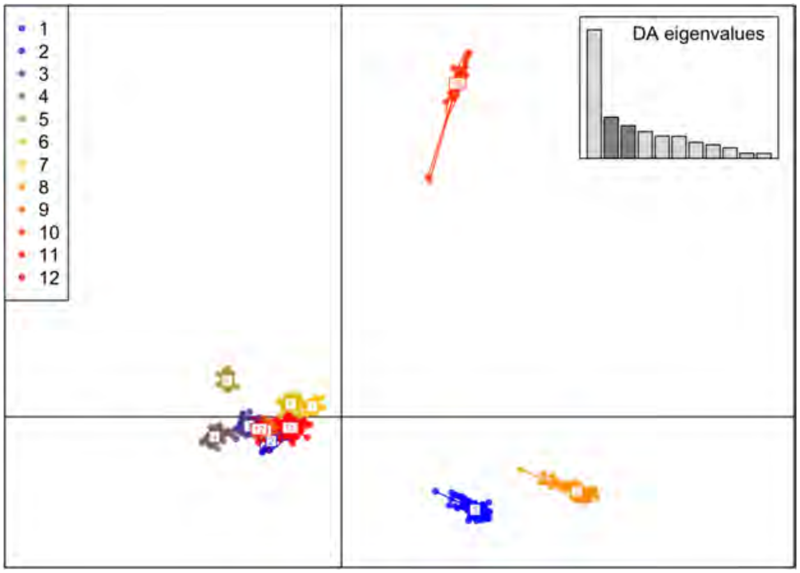


```
scatter(dapc, xax = 3, yax = 4, clab = 0.5,
        legend = T, posi.leg = "topleft",
        scree.da = T, posi.da = "topright")
```

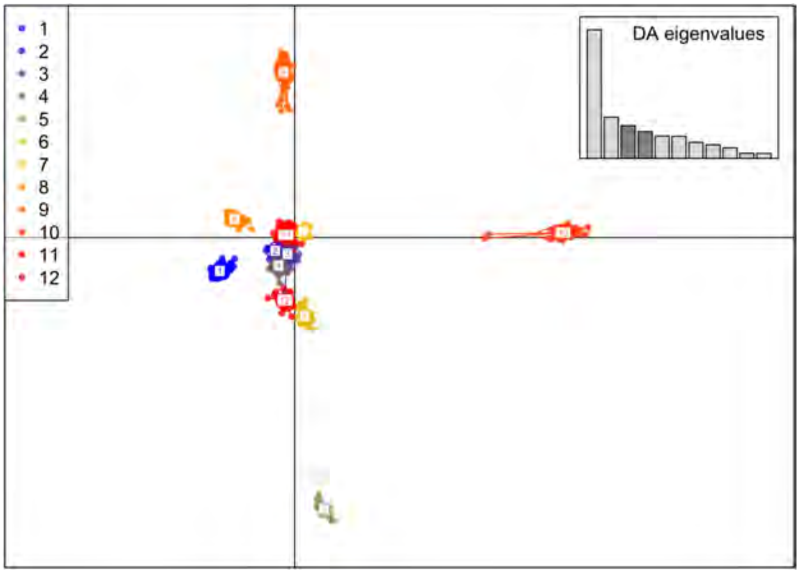


```
scatter(dapc, xax = 4, yax = 5, clab = 0.5,
        legend = T, posi.leg = "topleft",
        scree.da = T, posi.da = "topright")
```

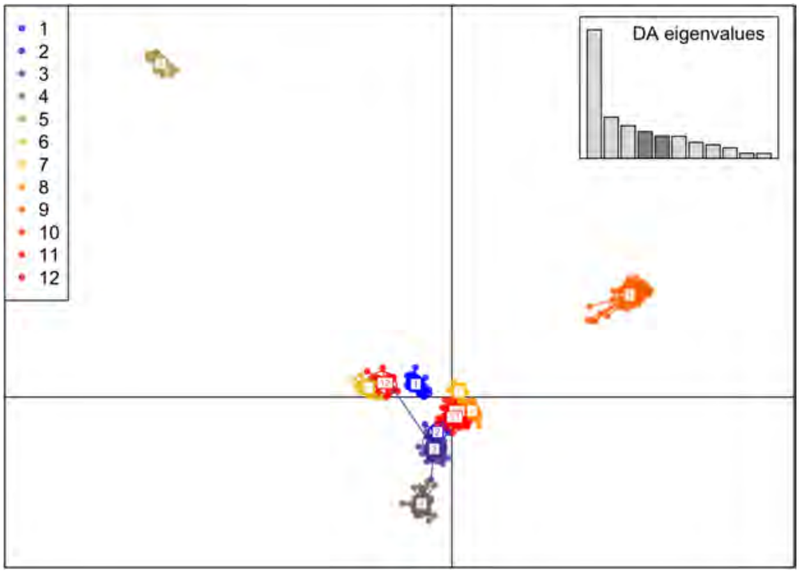

```
scatter(dapc, xax = 5, yax = 6, clab = 0.5,
        legend = T, posi.leg = "topleft",
        scree.da = T, posi.da = "topright")
```

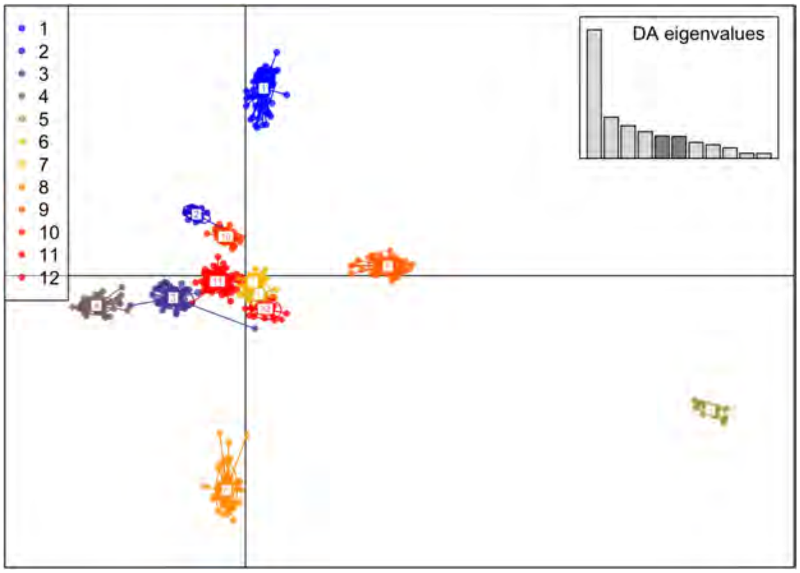


```
scatter(dapc, xax = 6, yax = 7, clab = 0.5,
        legend = T, posi.leg = "topleft",
        scree.da = T, posi.da = "topright")
```

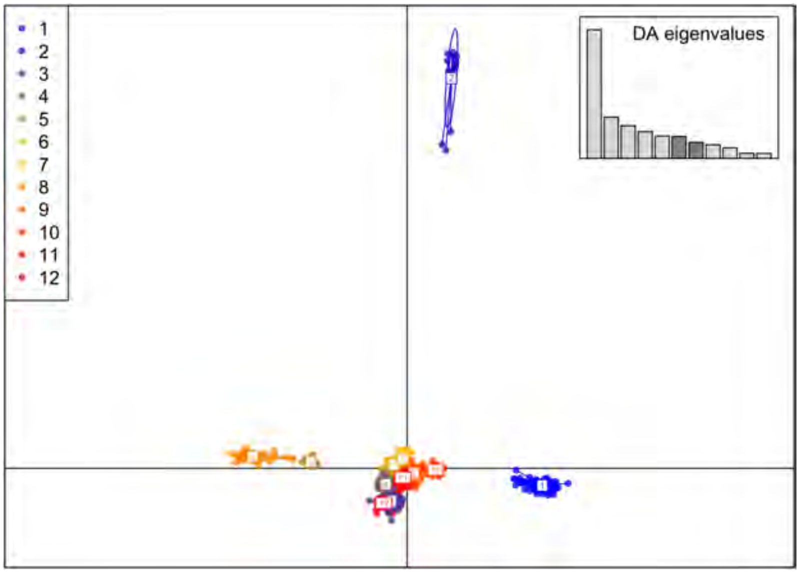


```
scatter(dapc, xax = 7, yax = 8, clab = 0.5,
        legend = T, posi.leg = "topleft",
        scree.da = T, posi.da = "topright")
```

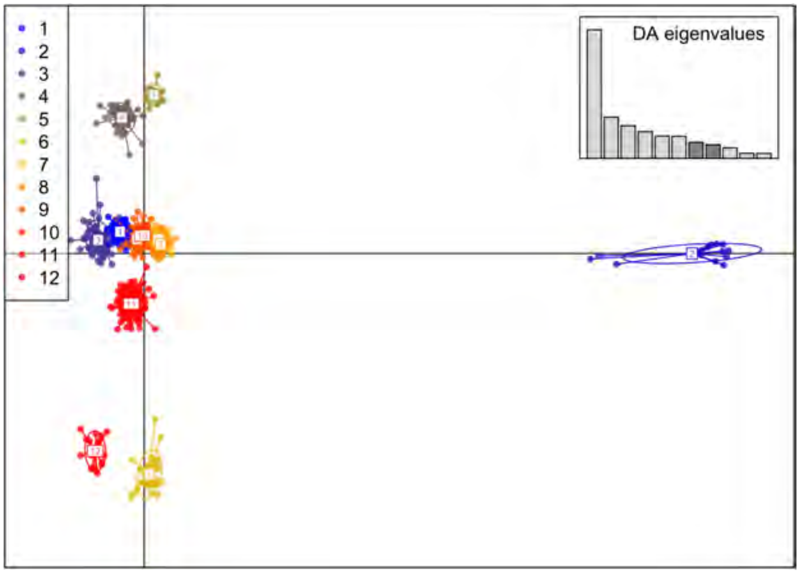

```
scatter(dapc, xax = 8, yax = 9, clab = 0.5,
        legend = T, posi.leg = "topleft",
        scree.da = T, posi.da = "topright")
```

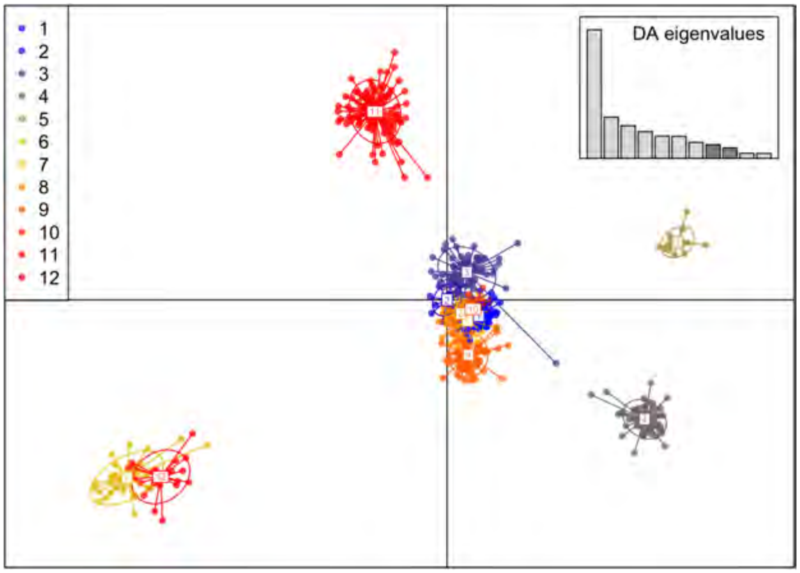


```
scatter(dapc, xax = 9, yax = 10, clab = 0.5,
        legend = T, posi.leg = "topleft",
        scree.da = T, posi.da = "topright")
```

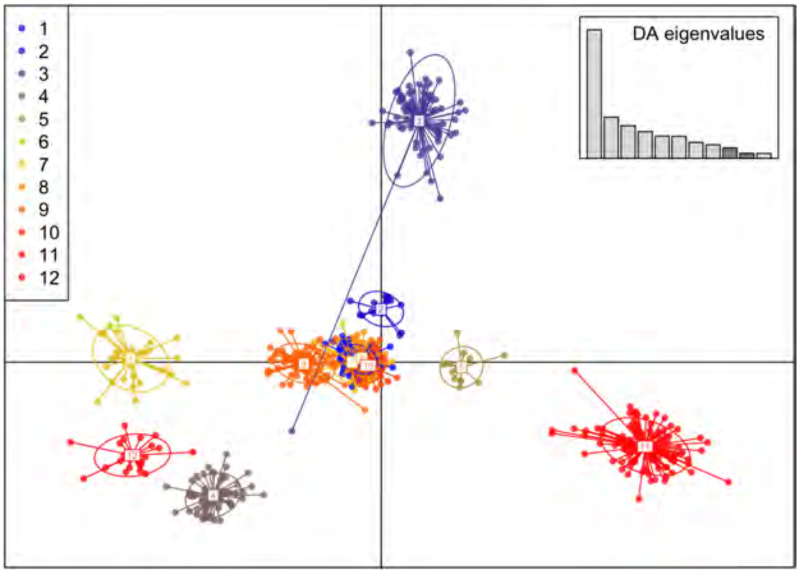


```
scatter(dapc, xax = 10, yax = 11, clab = 0.5,
        legend = T, posi.leg = "topleft",
        scree.da = T, posi.da = "topright")
```

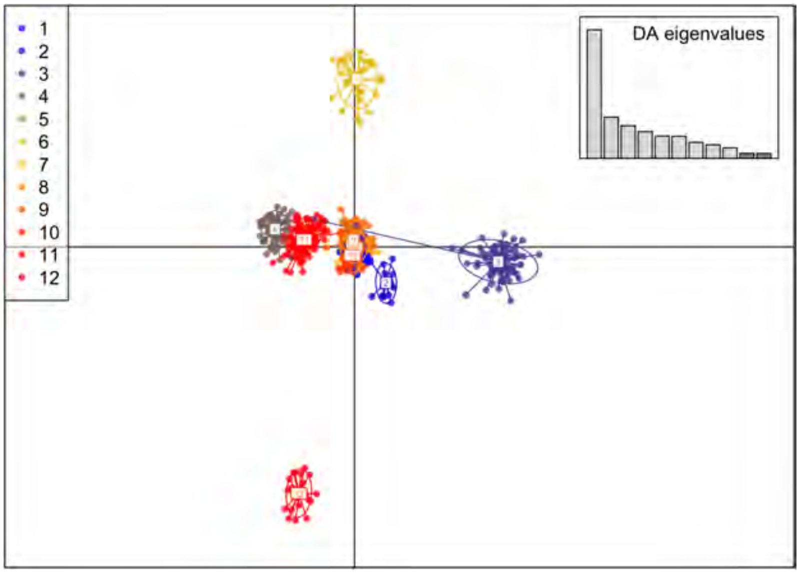

Three species DAPC

First I define the species by their sampling streams, then follow the DAPC procedure above but with the clusters already forced as the three species. I also look at the loci with the largest loadings on both DFs, and find that there are zero fixed differences between species groups. The high number of PCs retained in this DAPC (and therefore possibly the one above) indicate that there may still be overfitting despite following best practices. Or it shows how powerful DAPC can be: since it maximizes between group differences, with this much data it is easy to find support for many different group arrangements.

```
robusta <- c("ARA", "ASH", "BLACK", "BUR", "CHC", "CON",
             "COTCAN", "EAGLE", "ECLR", "FOR", "FRAN", "MCG",
             "SMITHC", "TRO", "UPP", "WTBV", "WLDR", "WCC")
intermedia <- c("BEAR", "BON", "CIEN", "DIX", "EAG", "HAR",
                "HSC", "IND", "LAR", "LSYC", "ODON", "RTD",
                "SAB", "SPR", "SYC", "TURK", "GILA", "WAL")
nigra <- c("BUZ", "EVERDE", "FC", "GOC", "GUN", "MAR", "ROC", "TONTO", "WTBM")

species <- ifelse(gila@pop %in% robusta, "robusta",
                  ifelse(gila@pop %in% intermedia, "intermedia", "nigra"))

prelim_sp_DAPC <- dapc(gila, species, var.contrib = T,
                    pca.select = "percVar", perc.pca = 90, pca.info = T,
                    n.da = 2)

a <- optim.a.score(prelim_sp_DAPC)$best
```

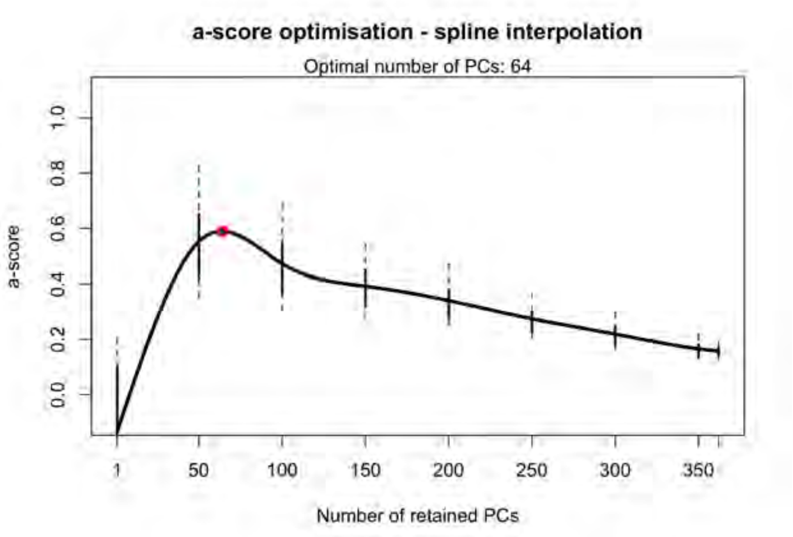


```
sp_DAPC <- dapc(gila, species, var.contrib = T,
             n.da = 2, n.pca = a, pca.info = T)

scatter(sp_DAPC, scree.da = F,
        scree.pca = T, posi.pca = "topright")
```

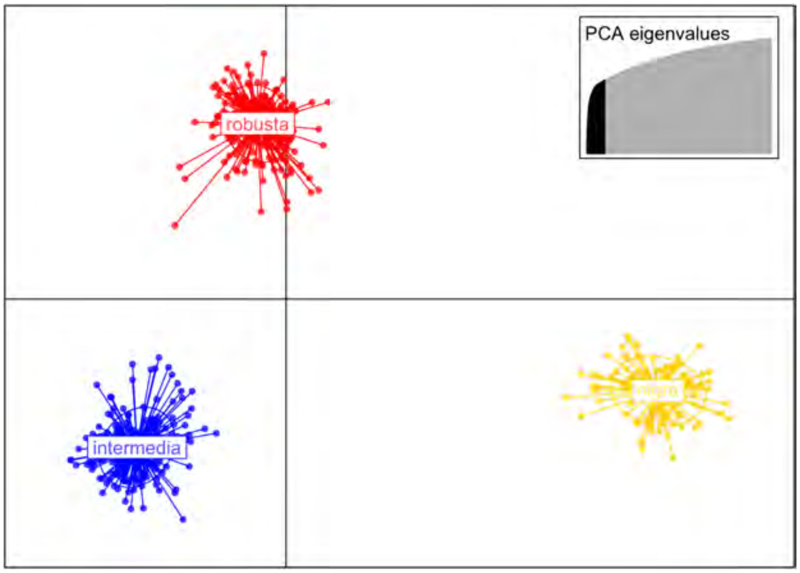


```
scatter(sp_DAPC, 1, 1)
```

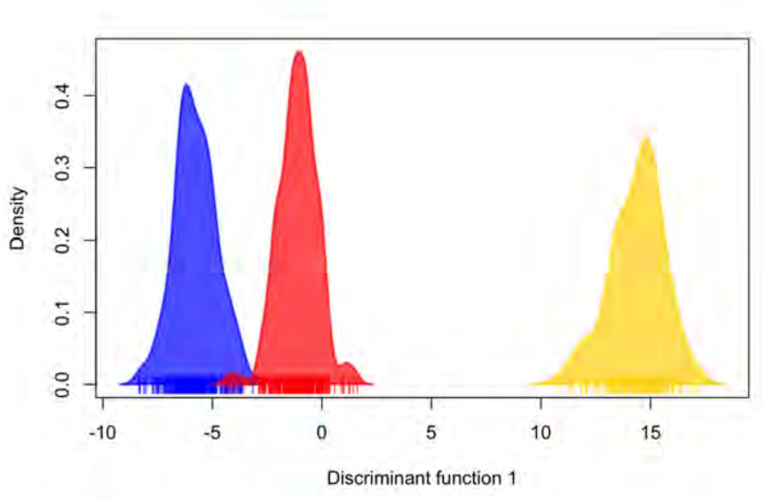


```
scatter(sp_DAPC, 2, 2)
```

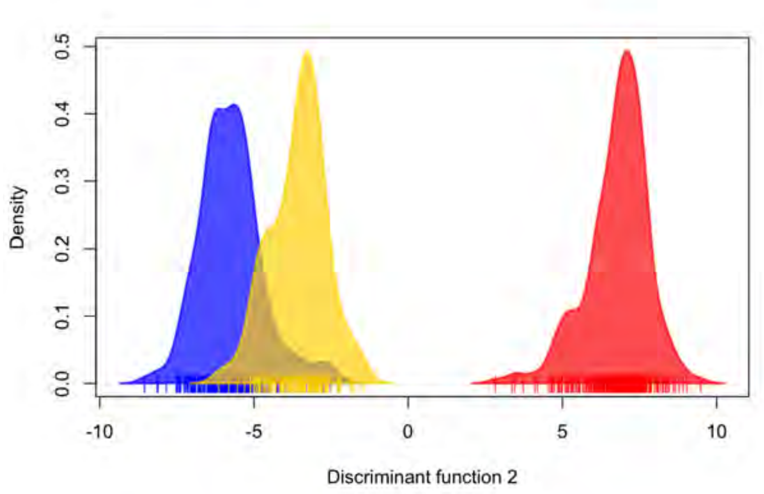


```
contrib <- loadingplot(sp_DAPC$var.contr, axis = 1, thres = 0.00145, lab.jitter = 0)
```

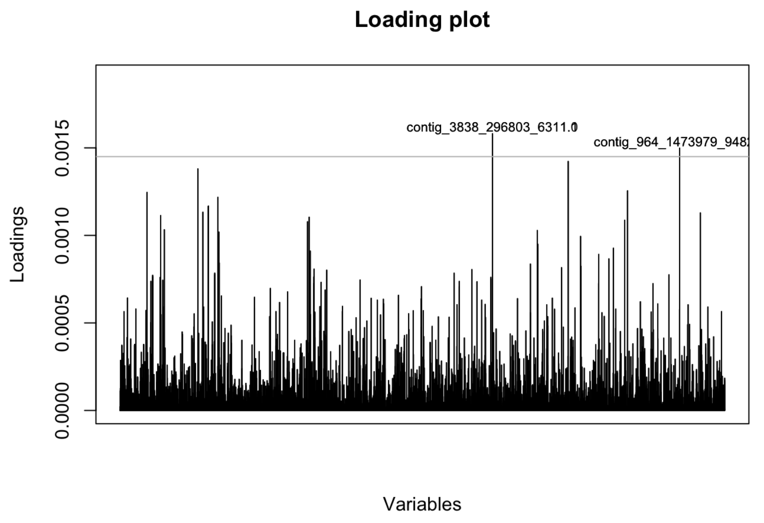


```
## Redo functions + lists by species instead of cluster
allele_freqs <- function(ind_mat) {
    apply(ind_mat, 2, function(e) tapply(e, species, mean, na.rm = TRUE))/2
}
freq_list <- lapply(loc_list, function(x) allele_freqs(tab(x)))

plot_freqs(freq_list$contig_3838_296803_6311)
```

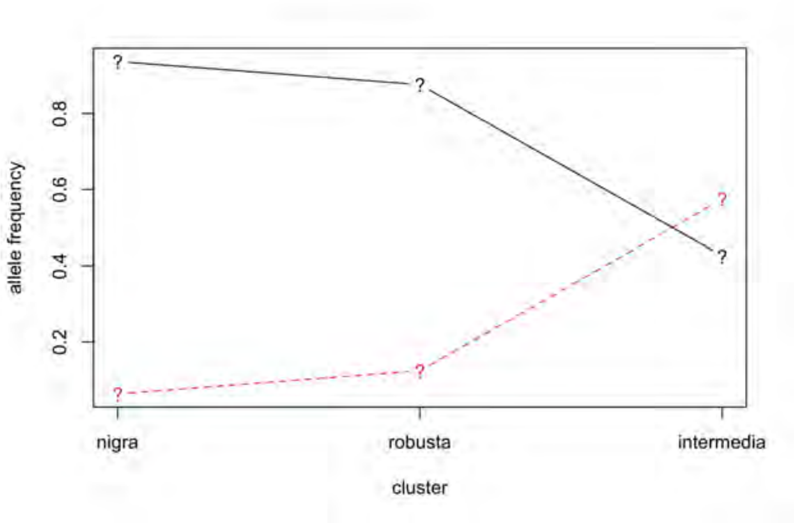


```
plot_freqs(freq_list$contig_964_1473979_94)
```

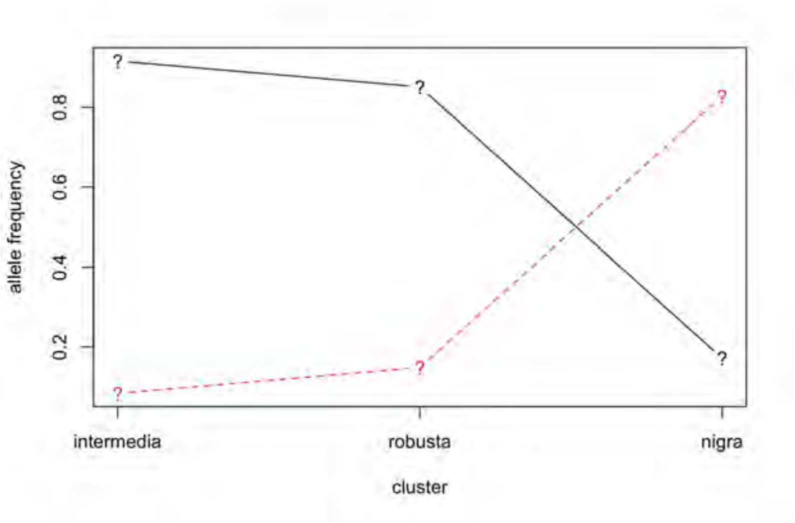


```
contrib <- loadingplot(sp_DAPC$var.contr, axis = 2, thres = 0.00135, lab.jitter = 0)
```

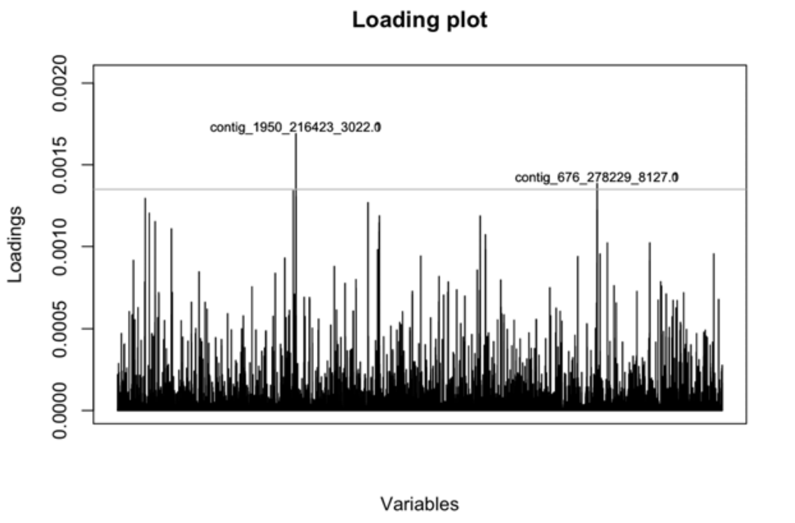


```
plot_freqs(freq_list$contig_1950_216423_3022)
```

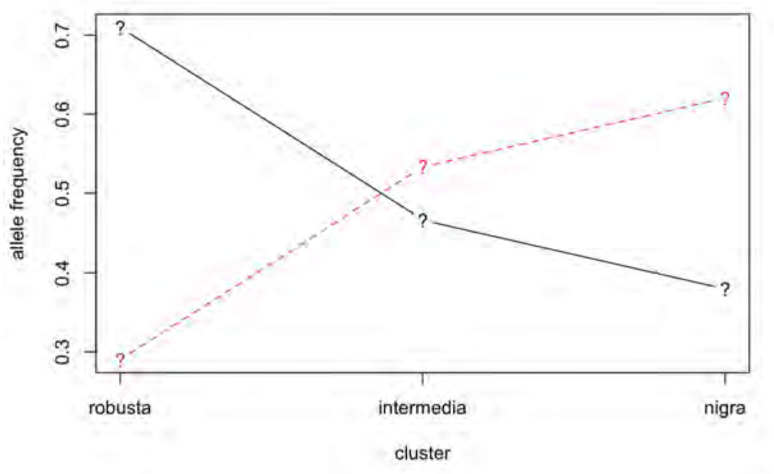


```
plot_freqs(freq_list$contig_676_278229_8127)
```

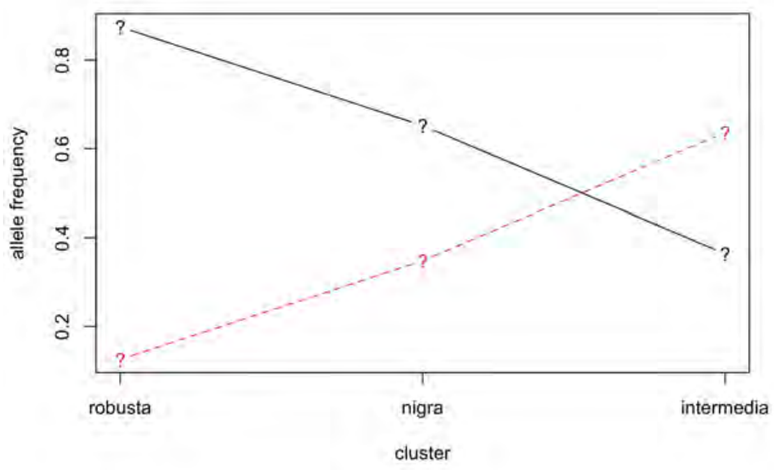


```
species_fixed_differences <- list()
for(a in seq_along(unique(species))) {
species_fixed_differences[a] <- list(names(which(!is.na(sapply(freq_list,
                                                               function(x) ifelse(x[a,1]==1 && sum(x[,1])==1 |
                                x[a,2]==1 && sum(x[,2])==1, x, NA))))))
}
total_fixed_diffs <- sapply(species_fixed_differences, length)
names(total_fixed_diffs) <- c("intermedia", "nigra", "robusta")
total_fixed_diffs
```

```
## intermedia      nigra    robusta
##          0          0          0
```

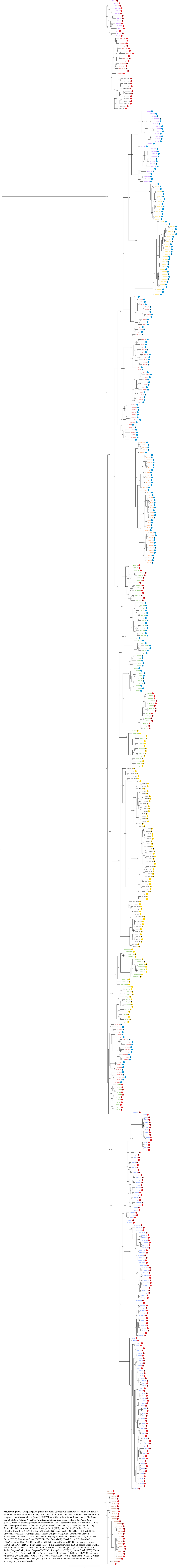

Modified Figure 2: Complete phylogenetic tree of the *Gila robusta* complex based on 10,246 SNPs for all individuals sequenced for this study. Site label color indicates the watershed for each stream location sampled: Little Colorado River (brown), Bill Williams River (blue), Verde River (green), Gila River (red), Salt River (black), Agua Fria River (orange), Santa Cruz River (yellow), San Pedro River (purple). Symbols following sample ID indicate taxonomic assignment to nominal taxa within the *Gila robusta* complex: *G. robusta* (red dot - R), *G. intermedia* (blue dot - I), *G. nigra* (mustard dot - N). Sample IDs indicate stream of origin: Aravaipa Creek (ARA), Ash Creek (ASH), Bear Canyon (BEAR), Black River (BLACK), Bonita Creek (BON), Burro Creek (BUR), Buzzard Roost (BUZ), Chevelon Creek (CHC), Cienega Creek (CIEN), Conger Creek (CON), Cottonwood Canyon (COTCAN), Dix Creek (DIX), Eagle Creek (EAG), Eagle Creek-below barrier (EAGLE), East Clear Creek (ECLR), East Verde River (EVERDE), Fort Rock (FOR), Fossil Creek (FC), Francis Creek (FRAN), Gordon Creek (GOC), Gun Creek (GUN), Harden Cienega (HAR), Hot Springs Canyon (HSC), Indian Creek (IND), Larry Creek (LAR), Little Sycamore Creek (LSYC), Marsh Creek (MAR), McGee Wash (MCG), O'Donnell Canyon (ODON), Red Tank Draw (RTD), Rock Canyon (ROC), Sabino Canyon (SAB), Smith Canyon (SMITHC), Spring Creek (SPR), Sycamore Creek (SYC), Tonto Creek (TONTO), Trout Creek (TRO), Turkey Creek (TURK), Upper Gila River (GILA), Upper Verde River (UPP), Walker Creek (WAL), Wet Beaver Creek (WTBV), Wet Bottom Creek (WTBM), Wilder Creek (WLDR), West Clear Creek (WCC). Numerical values on the tree are maximum likelihood bootstrap support for each node.
